# Identification of miRNA signature for predicting the prognostic biomarker of squamous cell lung carcinoma

**DOI:** 10.1371/journal.pone.0264645

**Published:** 2022-03-15

**Authors:** Huanqing Liu, Tingting Li, Chunsheng Dong, Jun Lyu

**Affiliations:** 1 Clinical Research Center, The First Affiliated Hospital of Xi’an Jiaotong University, Xi’an, Shaanxi, China; 2 Department of Clinical Research, The First Affiliated Hospital of Jinan University, Guangzhou, Guangdong, China; 3 Department of Pharmacy, Xi’an Chest Hospital, Xi’an, Shaanxi, China; 4 School of Computer Science, Shaanxi Normal University, Xi’an, Shaanxi, China; All India Institute of Medical Sciences, INDIA

## Abstract

As explorations deepen, the role of microRNAs (miRNAs) in lung squamous cell carcinoma (LUSC), from its emergence to metastasis and prognosis, has elicited extensive concern. LUSC-related miRNA and mRNA samples were acquired from The Cancer Genome Atlas (TCGA) database. The data were initially screened and pretreated, and the R platform and series analytical tools were used to identify the specific and sensitive biomarkers. Seven miRNAs and 15 hub genes were found to be closely related to the overall survival of patients with LUSC. Determination of the expression of these miRNAs can help improve the overall survival of LUSC patients. The 15 hub genes correlated with overall survival (OS). The new miRNA markers were identified to predict the prognosis of LUSC. The findings of this study offer novel views on the evolution of precise cancer treatment approaches with high reliability.

## Introduction

Lung carcinoma, one of the most serious malignancies, is the primary cause of cancer-related deaths worldwide [1]. Recent statistics show that 2.21 million people were diagnosed with lung carcinoma in 2020, and 1.8 million died from lung cancer, which has the highest mortality rate among all cancer types [2]. Radiation therapy and targeted therapy do not considerably improve the patient’s survival, and the overall 5-year survival rate remains below 20% [3]. Lung carcinoma has a serious impact on human health and has become a public health problem. Lung squamous carcinoma (LUSC) is a common histologic subtype of lung cancer characterized by atypical early symptoms and inherent resistance to radiation and chemotherapy. The lack of appropriate targeted drugs has led to a poorer prognosis of LUSC compared with lung adenocarcinoma [4]. The prognosis of LUSC still relies mainly on the histopathological diagnosis and tumor staging. However, traditional methods are unable to accurately assess the prognosis of patients with LUSC. Therefore, identifying the prognostic markers and establishing new and reliable prognostic models are crucial for enhancing the quality of life, prognosis, and overall survival (OS) of LUSC patients.

MicroRNAs (miRNAs), transcribed by type II and III RNA polymerases, can bind to the 3’ untranslated regions of mRNAs and prevent mRNA translation [5]. It regulates 30% of mRNA and plays a role in cell growth, apoptosis, differentiation, cellular proliferation, and stress response. miRNAs are usually located in cancer-associated genomic regions, comprising fragile sites, together with the regions of frequent LOH, deletion, amplification, and translocation, most of which are amplified or deleted in patients with cancer [6]. Measurement of the levels of circulating miRNA in patients’ serum may be a simple and noninvasive method for diagnosing early-stage cancer. In 2015, researchers identified five miRNA levels in serum, and their elevated levels could be used to diagnose lung cancer [7]. miRNAs are involved in carcinoma progression and act as suppressors or promoters [8]. MiR-10b has an antitumor function in HPV-positive cervical carcinoma by suppressing TIAM1 [9]. The overexpression of miR-145, miR-21, and miR-10b is significantly associated with inferior prognosis in patients with gastric carcinoma [10]. Some previous studies have also evaluated the role of miRNAs in lung cancer. Abnormalities of miR-182, miR-205, miR-183, and miR-96 have been observed in lung carcinoma samples. The overexpression of miR-125b can promote the metastasis of non-small cell lung carcinoma (NSCLC) cells, while decreased miR-125b reduced the cell migration process [11]. Nevertheless, a few molecular markers can evaluate carcinoma-associated miRNAs in a systematic manner and predict the OS or immunotherapy responses. To date, different kinds of databases have been established to store various data related to miRNAs, mRNAs, etc, which contain comprehensive information [12]. Global gene expression data were utilized to analyze the relationship between carcinoma-related miRNA expression and clinical prognosis in LUSC patients. Chen et al. [13] used the data collected from Gene Expression Omnibus (GEO), The Cancer Genome Atlas (TCGA), literature screening, and real-time quantitative real-time PCR (RT-qPCR) analyses to determine the clinical role of miR-144-3p in NSCLC. The TCGA database was mainly used for cancer bioinformatics research. Hamilton et al. [14] discovered a superfamily of oncogenic miRNAs jointly regulated by cancer by combining the TCGA database and the microRNA database. Li et al. [15] also found a new tumor target gene of LUSC by comparing genes in the TCGA and GEO databases. In our current study, RNA sequencing (RNA-seq) and miRNA sequencing (miRNAs-seq) data were obtained from TCGA database to establish an miRNA-based prognostic signature; then, the differentially expressed miRNAs and mRNAs in LUSC samples and adjacent tissue samples were analyzed. A protein-to-protein interaction (PPI) network was also constructed. The identified miRNAs and related genes may offer a novel theoretical foundation for LUSC prognosis and for developing a targeted therapy.

## Materials and methods

### Data download and screening

**Fig 1:**
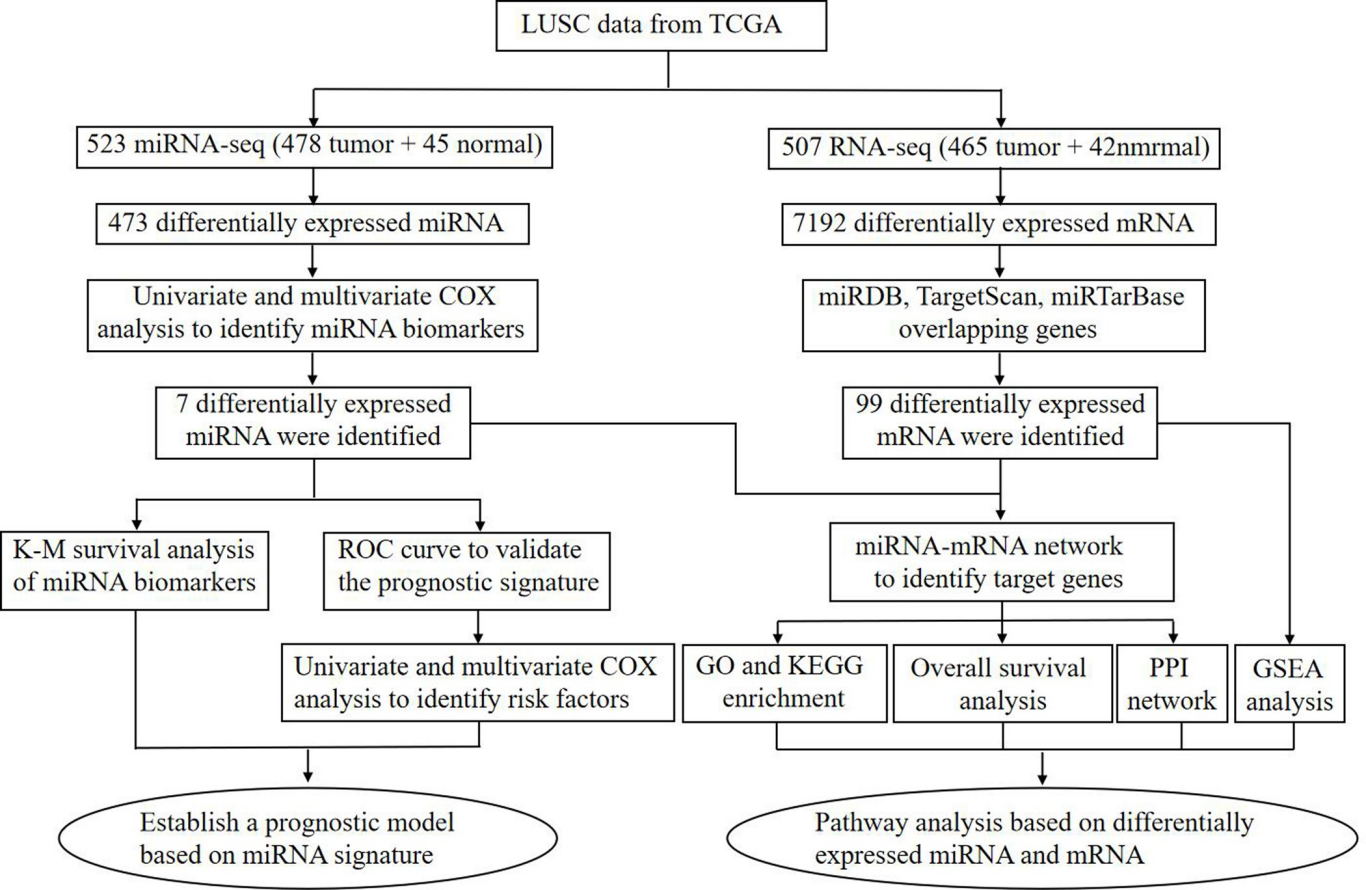
Flowchart of the bioinformatics analysis of LUSC data from TCGA databases.

TCGA database (https://cancergenome.nih.gov/) contains standardized clinical data on various cancer types and gene and miRNA expression data. The RNA-seq and miRNA-seq data of LUSC patients were downloaded from TCGA. We obtained 507 RNA-seq (42 normal and 465 tumor) samples and 523 miRNA-seq (45 normal and 478 tumor) samples. The clinicopathological data, including sex, age, staging status, TMN type, survival state, and length of survival, were collected. These data are shown in Table 1. The clinical information and data from LUSC patients downloaded from TCGA database were integrated and analyzed through bioinformatics analysis, as shown in Fig 1. TCGA is a public database. Users can download relevant data for free that can be used for conducting research and publish relevant articles. Our study is based on open-source data; no ethical issues were raised during the conduct of this study.

**Table 1:** Characteristics of LUSC patients in TCGA database.

variable	Number of samples
Gender	
Male/Female	373/131
Age at diagnosis	
≤65/>65/NA	190/305/9
Stage	
Ⅰ/Ⅱ/Ⅲ/Ⅳ/NA	245/163/85/7/4
T	
T0/T1/T2/T3/T4/NA	0/114/295/71/24/0
M	
M0/M1//MX/NA 184/1/326	414/7/79/4
N	
N0/N1/N2/N3//NX/NA	320/133/40/5/6

### Prognostic signature construction and nomogram development indicators based on the expression of miRNA

The expression profiles of mRNA and miRNA were standardized using edgeR (R package). mRNA and miRNAs with different expression levels showed a false discovery rate (FDR) of <0.05 and a |log FC| value of ≥1. The MiRNAs were classified into two groups using caret (R package), which creates data partition functionality: a training set and a testing set. The prognostic value of miRNAs was initially assessed using univariate Cox regression. Data of the MiRNAs obtained from the univariate Cox regression model and clinical factors were used in the multivariate Cox proportional hazard regression model. Only miRNA and clinical factors (p < 0.05) in the univariate and multivariate COX analyses could be considered as prognostic factors for LUSC. The selection of significant miRNA levels was performed using univariate and multivariate COX regression analyses, and the result was used as the model miRNA. The prognostic indicators were as follows: risk score = (coefficient miRNA1 ×expression of miRNA1) + (coefficient miRNA2 ×expression of miRNA2) + +(coefficient miRNAn × expression miRNAn). Time-dependent receiver operating characteristic (ROC) curves were obtained using the R-SURVIVvalroc software package to assess the specificity and sensitivity of the prognostic characteristics based on the levels of miRNA expression. Kaplan Meier survival was used to analyze the impact of selected miRNAs and mRNAs on the strength of the survival association in LUSC patients and to compare the survival of high-risk and low-risk patients.

### Enrichment analyses of target genes

We obtained the data of candidate target genes for prognostic miRNAs from miRDB (http://www.mirdb.org/miRDB/), TargetScan (http://www.targetscan.org/), and miRTarBase. At least two overlapping genes were selected from the three online resources as miRNA target genes. The interaction networks between target genes and miRNA were visualized using Cytoscape; the enrichment of Gene Ontology Analysis (GO) and Kyoto Encyclopedia of Genes and Genomes (KEGG) was conducted using the David Database. Compared with a common KEGG analysis, gene set enrichment analysis (GSEA) (http://www.gsea-msigdb.org/gsea/index.jsp) was used to identify the upregulation and downregulation relationship of individual gene enrichment pathways in a certain disease. All lung squamous cell carcinoma genes were enriched using the GSEA software, and the pathways with |NES|≥ 1.0, NOM p ≤ 0.05, and FDR q-Val ≤ 0.25 were selected for visualization.

## Results

### Establishment of the prognostic signature miRNA

**Fig 2:**
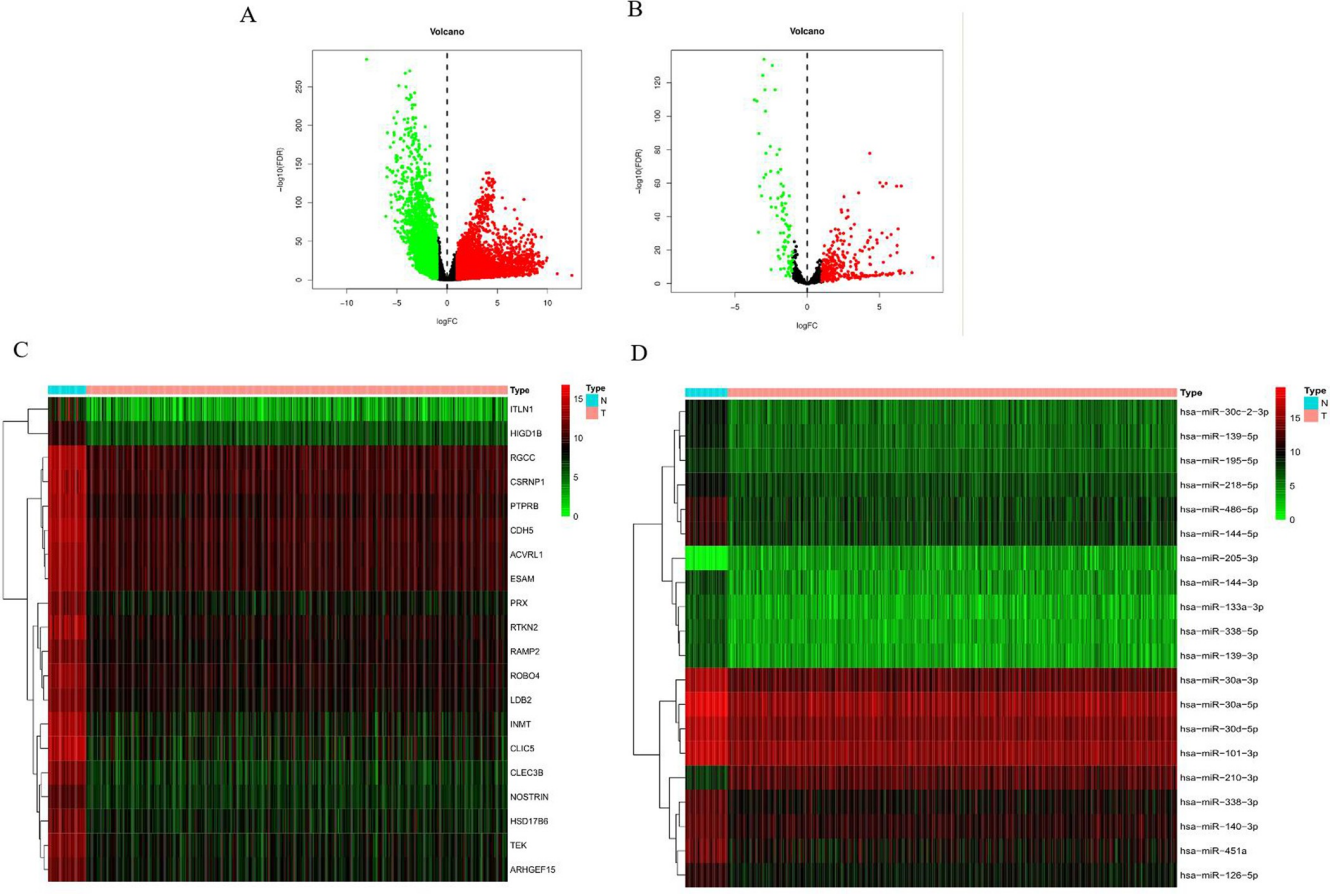
Volcano and heat maps of the top 20 upregulated/downregulated differentially expressed genes or miRNAs. A, C: mRNA; B, D: miRNAs.

**Fig 3:**
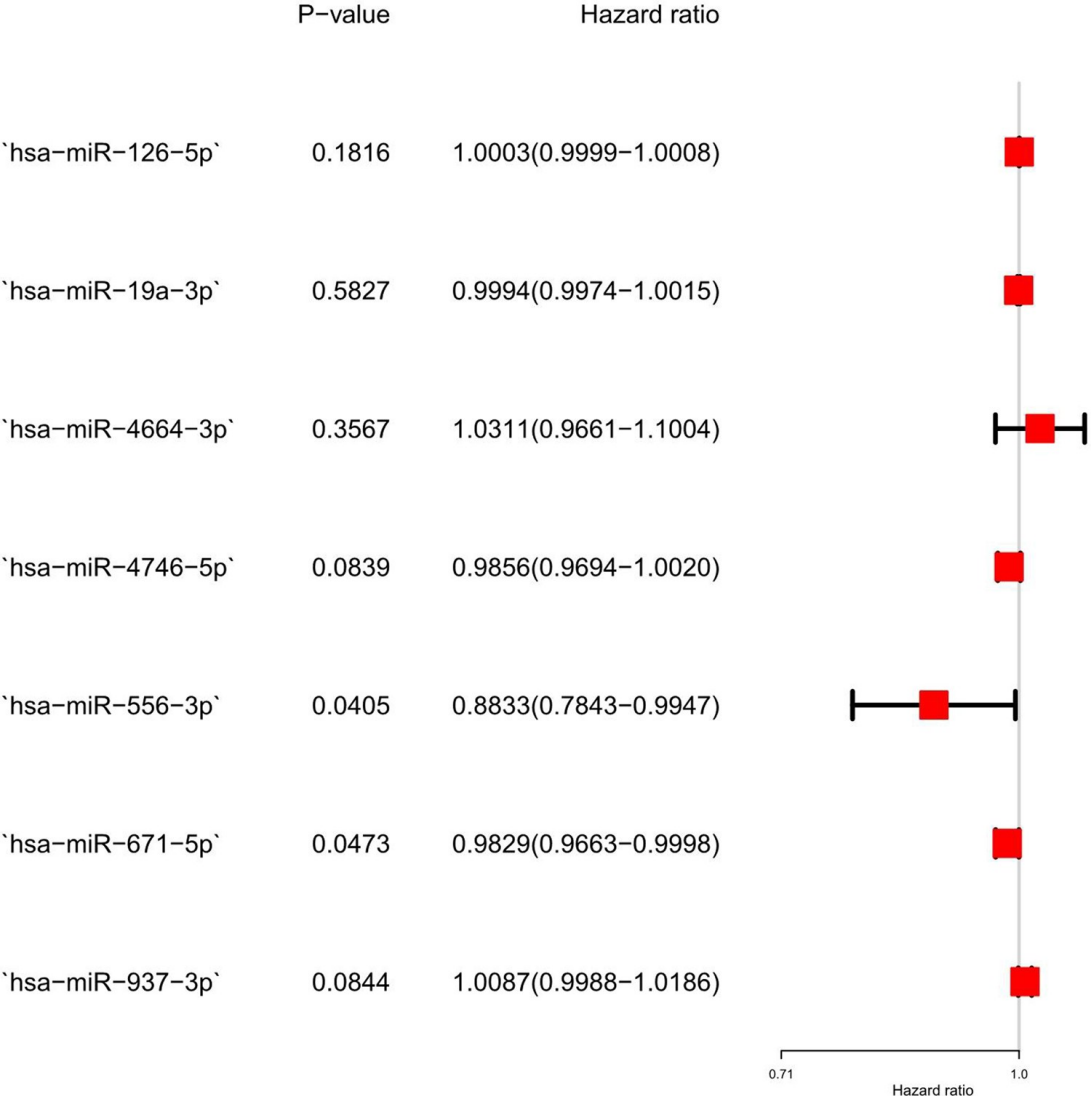
Multivariate Cox analysis to identify differentially expressed miRNAs.

TCGA database had 7,192 mRNAs with different expression levels (4,311 upregulated and 2,881 downregulated, S1 Table) and 473 miRNAs with different expression levels (305 upregulated and 84 downregulated, S2 Table). Fig 2A and 2C shows the volcanic map and heat map of the mRNA expression of the top 20 upregulated and downregulated genes, respectively. Fig 2B and 2D show the volcano plot of miRNAs with different expression levels and the heatmap of the top 20 miRNAs undergoing upregulation/downregulation. For prognostic screening of miRNAs, Cox regression analysis was used to verify the characteristics of miRNAs with different expression levels. Results of the univariate Cox analysis are shown in S3 Table. Ultimately, seven differentially expressed miRNAs (hsa-miR-19a-3p, hsa-miR-126-5p, hsa-miR-556-3p, hsa-miR-671-5p, hsa-miR-937-3p, hsa-miR-4664-3p, and hsa-miR-4746-5p) were selected in the training group as independent prognostic factors. According to the multivariate Cox analysis outcomes, two miRNAs (hsa-miR-556-3p and hsa-miR-671-5p) could be investigated in the future (p < 0.05) (Fig 3). This model was expressed using the following formula: risk score = (−0.0007 × expression hsa-miR-19a-3p) + (0.0002 × expression hsa-miR-126-5p) + (−0.0873 × expression hsa-miR-556-3p) + (−0.0127 × expression hsa-miR-671-5p) + (0.0092 × expression hsa-miR-937-3p) + (0.0689× expression hsa-miR-4664-3p) + (−0.0181 × expression hsa-miR-4746-5p). Additionally, the risk score for each patient in this cohort was calculated. The cohort should then be divided into high-risk and low-risk groups, using the median risk score as the cutoff value for the training set and the test set.

### Survival outcomes and multivariate examination

**Fig 4:**
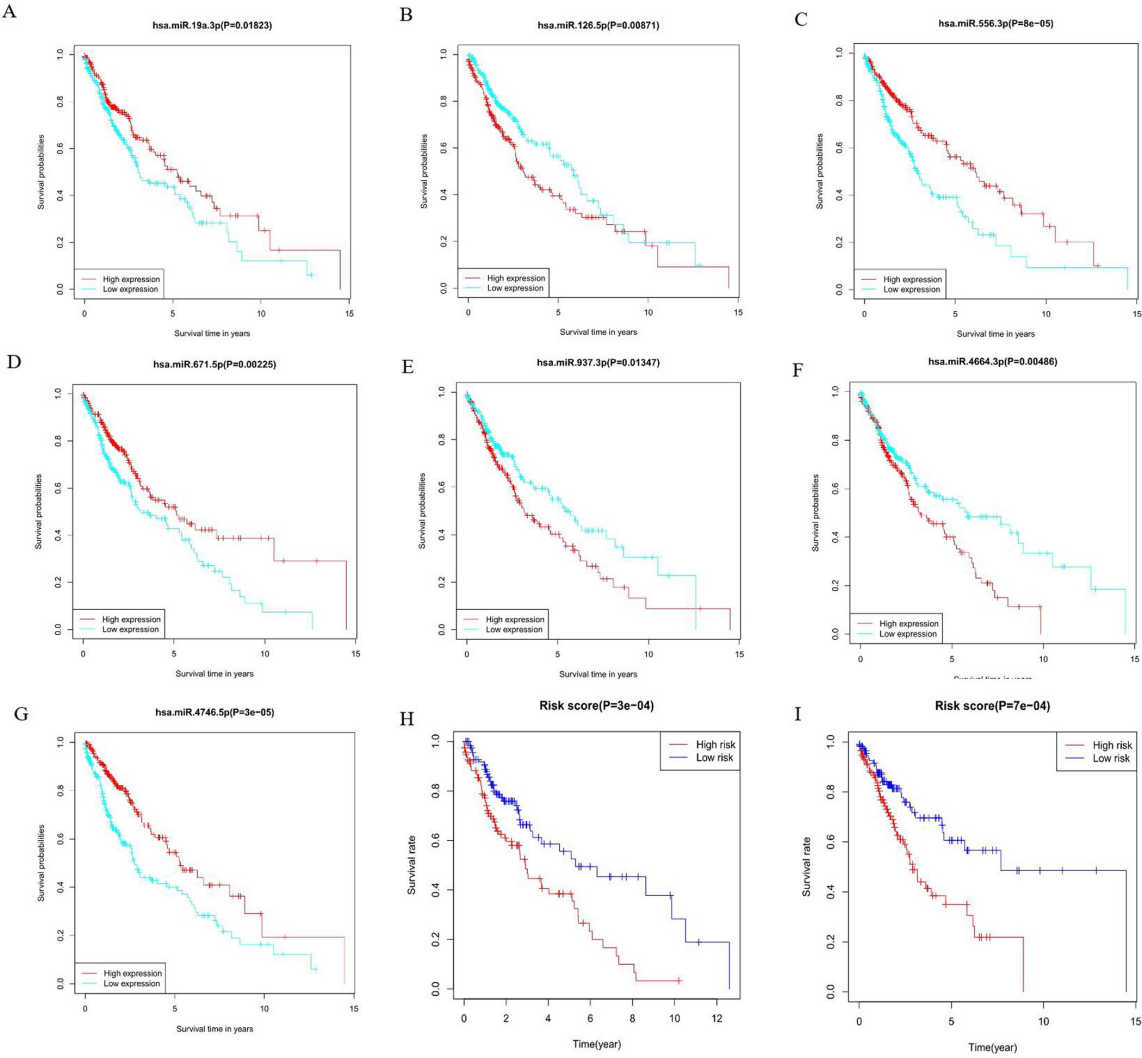
Overall survival analysis of 7 miRNA, the training set and testing set. A-G: miRNAs; H: Training set; I: Testing set.

**Fig 5:**
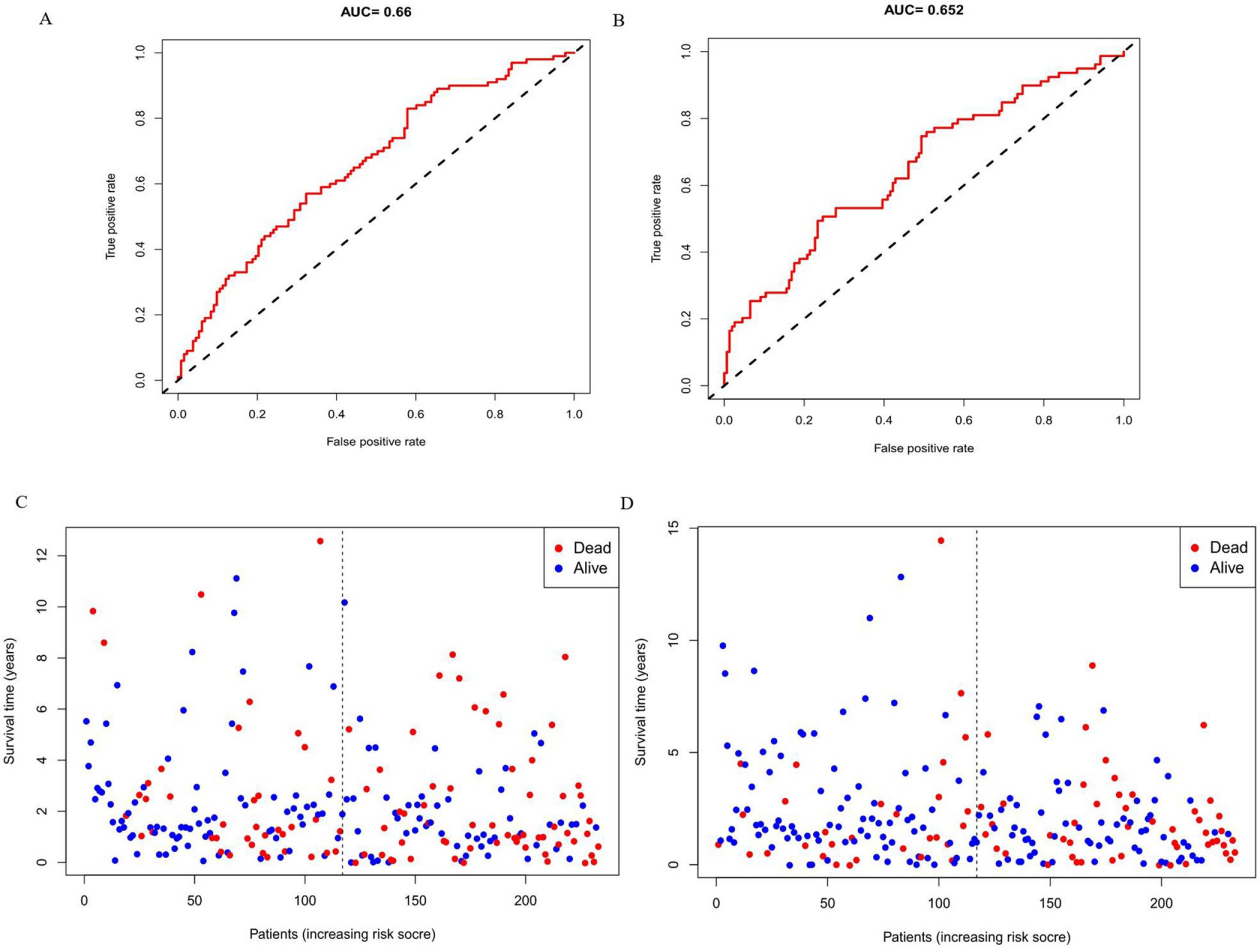
ROC curves and risk survival status of patients in the training set and testing set. A: ROC in training set; B: ROC in testing set; C: Survival status in the training set; D: Survival status in testing set.

**Fig 6:**
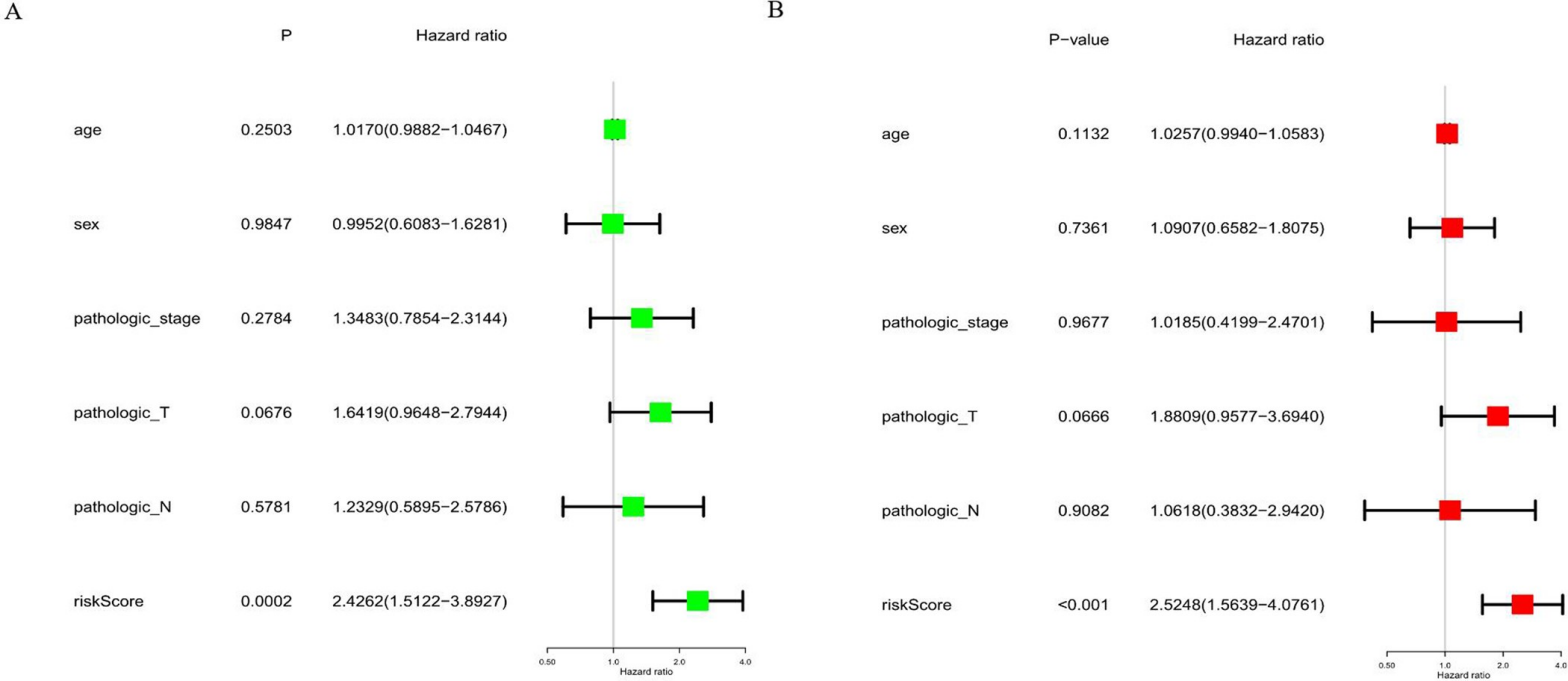
Univariate and multivariate Cox analyses to identify the risk factors. A: Univariate; B: Multivariate.

In this study, the Kaplan–Meier curve was used to analyze the effect of the expression of the seven miRNAs on the intensity of the correlation with survival. The expression levels of has-miR-19a-3p (p = 0.01823), hsa-miR-126-5p (p = 0.00871), hsa-miR-556-3p (p = 8e−05), hsa-miR-671-5p (p = 0.00225), hsa-miR-937-3p (p = 0.01347), hsa-miR-4664-3p (p = 0.00486), ahashsa-miR-4746-5p (p = 3e−05) significantly affected the OS (Fig 4A–4M). Additionally, Kaplan-Meier curves were used for these two cohorts to detect the prediction value of the molecular signatures of these seven miRNAs. With regard to the training and testing cohorts, inferior survival outcomes were observed in the high-risk group than in the low-risk group (p = 3e−04; and p = 7e−04, respectively). These ROC curves were used to investigate whether the expression patterns of miRNAs related to survival were capable of the early prediction of LUSC. Results showed that the area under the curve (AUC) of the training set was 0.66, while that of the testing set was 0.652, indicating that this prognostic model was moderately sensitive and specific. The status plot of the risk survival of patients showed an increase in mortality with an increasing risk score for patients (Fig 5). To develop a prognostic model based on 7-miRNA, univariate and multivariate Cox analyses were used to identify the risk factors. Based on the characteristics of the seven miRNAs, the risk score (hazard ratio = 2.5248, 95% confidence interval = 1.5639−4.0761, p < 0.001) could function as an independent prognostic indicator for OS (Fig 6).

### MiRNA target gene analysis

**Fig 7:**
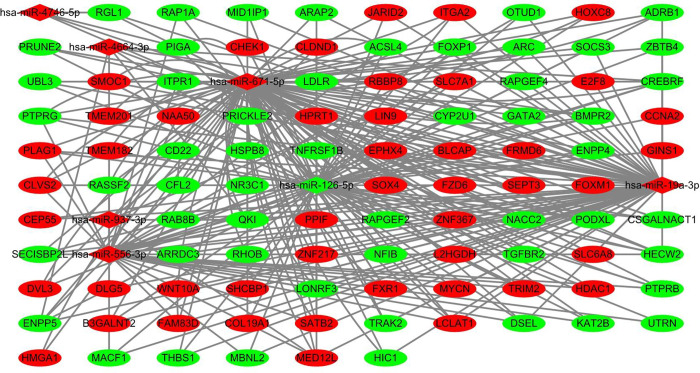
Potential association between microRNA and target genes. Red dot indicates upregulated, and green dot means downregulated.

**Fig 8:**
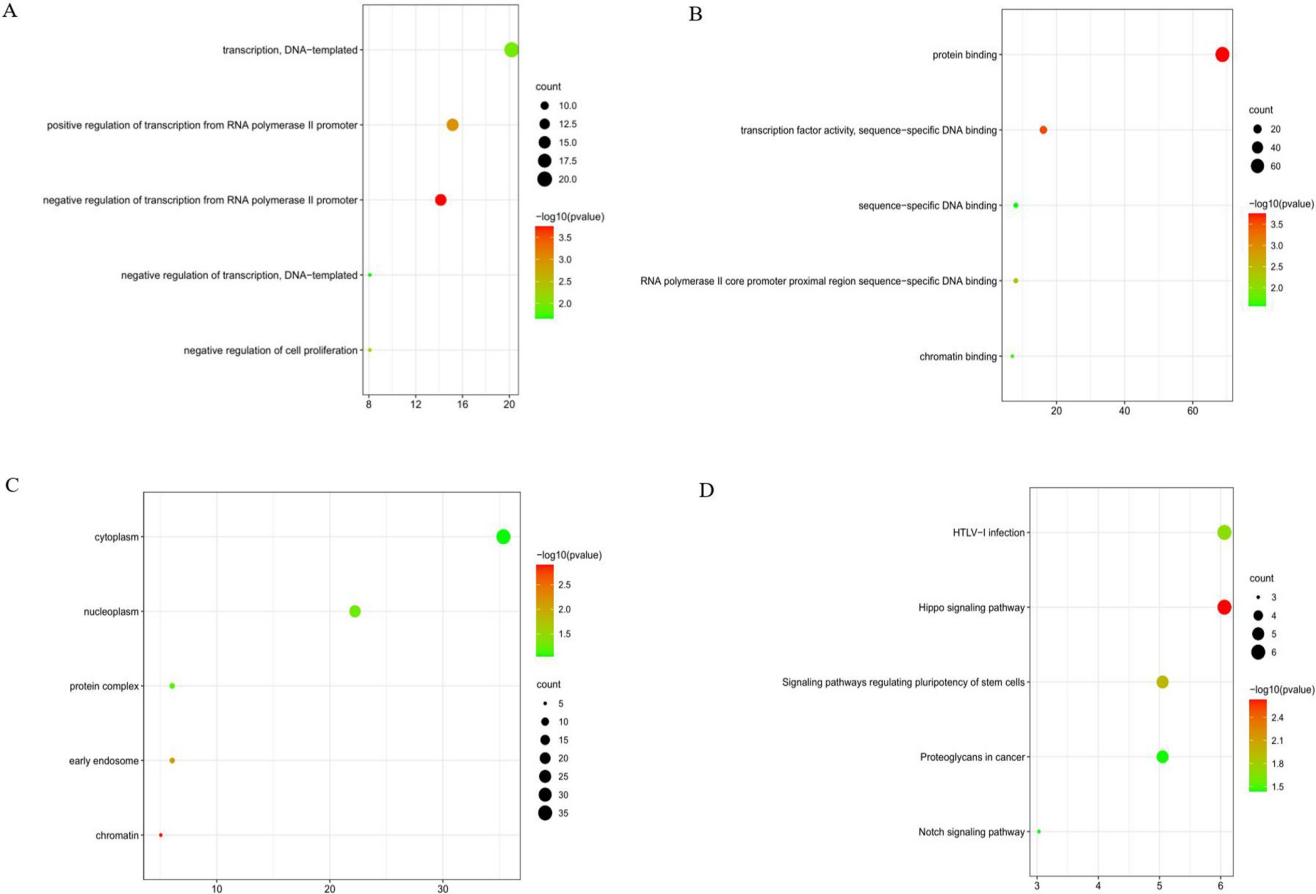
GO and KEGG analyses of target genes.

**Fig 9:**
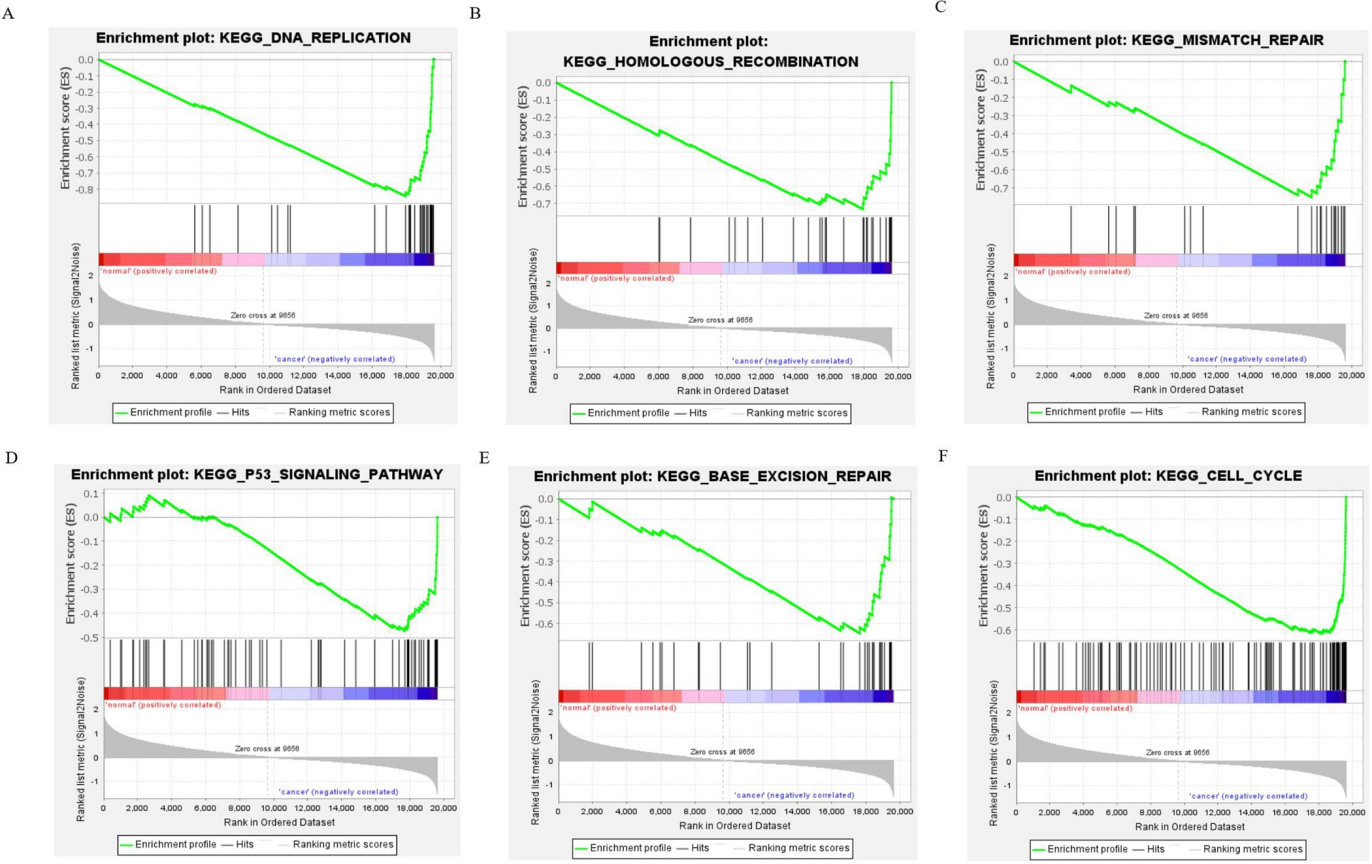
GSEA analysis of the differentially expressed mRNA.

The online database was utilized to predict the target genes of the seven miRNAs, and data of 99 genes were obtained (S4 Table). Cytoscape was used to investigate the potential associations between miRNAs and the target genes. According to the results shown in Fig 7, hsa-miR-126-5p possessed the greatest number of nodes within this network. GO enrichment and KEGG pathway analyses were performed to evaluate the biological functions of the target genes. GO analyses showed several alterations in the biological processes of the target genes, such as ameboidal-type cellular migration, positive regulation of neurogenesis, and regulation of neuron projection development. Molecular function analysis showed that the target genes were primarily involved in adrenergic receptor binding. Cell component enrichment analysis indicated that the genes were mainly enriched in early endosomes. KEGG pathway analysis revealed that the Hippo signaling pathway and human T-cell leukemia virus type I (HTLV-I) infections were the main enrichment pathways of the target genes (Fig 8 and S5 Table). Pathway enrichment analysis was performed for all differentially expressed genes in LUSC patients using GSEA software, and the results showed that the genes were significantly associated with base excision repair, cell cycle, DNA replication, homologous recombination, mismatch repair, and p53 signaling pathway (Fig 9).

### Overall survival analysis of miRNA target genes

**Fig 10:**
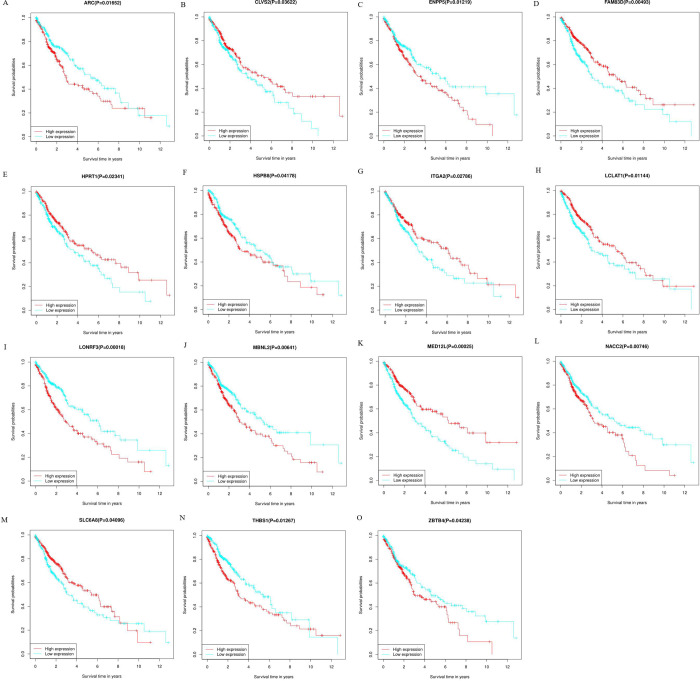
Overall survival analysis of the identified target genes and the protein-protein interaction (PPI) network. A-O: mRNA.

**Fig 11:**
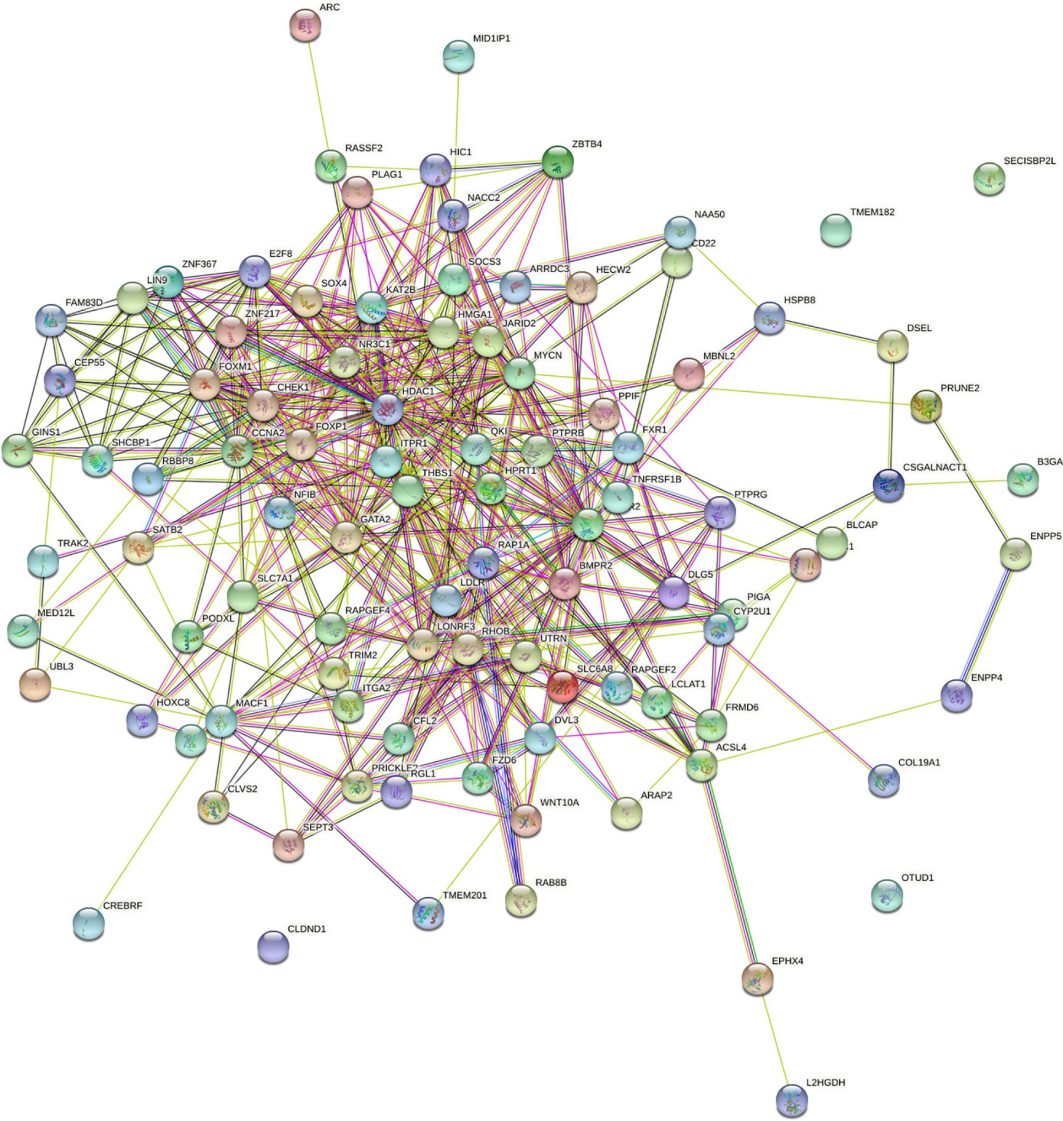
PPI network of LUSC related target genes.

Fifteen genes were identified by analyzing the influence of target gene expression on patient’s survival: ARC (p = 0.01652), CLVS2 (p = 0.03622), ENPP5 (p = 0.01219), FAM83D (p = 0.00493), HPRT1 (p = 0.02341), HSPB8 (p = 0.04178), ITGA2 (p = 0.02786), LCLAT1 (p = 0.01144), LONRF3 (p = 0.00018), MBNL2 (p = 0.00641), MED12L (p = 0.00025), NACC2 (p = 0.00746), SLC6A8 (p = 0.04096), THBS1 (p = 0.01267), and ZBTB4 (p = 0.04238), all of which had an apparent impact on OS (Fig 10A–10O). Establishment of the PPI network revealed two hub genes (CCNA2 and RAP1A) (Fig 11). The names, full names, and functions of these target genes are listed in Table 2.

**Table 2:** Functional roles of 15 hub genes.

No.	Gene symble	Full name	Function
1	*ARC*	Activity-regulated cytoskeleton-associated protein	It plays a role in the regulation of cell morphology and cytoskeletal organization.
2	*CLVS2*	Clavesin-2	It is required for normal morphology of late endosomes and/or lysosomes in neurons. Binds phosphatidylinositol 3,5-bisphosphate.
3	*ENPP5*	Ectonucleotide pyrophosphatase/phosphodiesterase family member 5	It may play a role in neuronal cell communication.
4	*FAM83D*	Protein FAM83D	It is probable proto-oncogene that regulates cell proliferation, growth, migration and epithelial to mesenchymal transition.
5	*HPRT1*	Hypoxanthine-guanine phosphoribosyltransferase	It plays a central role in the generation of purine nucleotides through the purine salvage pathway.
6	*HSPB8*	Heat shock protein beta-8	It displays temperature-dependent chaperone activity.
7	*ITGA2*	Integrin alpha-2	It is responsible for adhesion of platelets and other cells to collagens, modulation of collagen and collagenase gene expression, force generation and organization of newly synthesized extracellular matrix.
8	*LCLAT1*	Lysocardiolipin acyltransferase 1	It acts as a remodeling enzyme for cardiolipin, a major membrane polyglycerophospholipid.
9	*LONRF3*	LON peptidase N-terminal domain and ring finger 3	It is a protein coding gene associated with ATP-dependent peptidase activity.
10	*MBNL2*	Muscleblind-like protein 2	It acts either as activator or repressor of splicing on specific pre-mRNA targets.
11	*MED12L*	Mediator of RNA polymerase II transcription subunit 12-like protein	It is a coactivator involved in the regulated transcription of nearly all RNA polymerase II-dependent genes.
12	*NACC2*	Nucleus accumbens-associated protein 2	It functions as a transcriptional repressor through its association with the NuRD complex.
13	*SLC6A8*	Solute Carrier Family 6 Member 8	It is required for the uptake of creatine in muscles and brain
14	*THBS1*	Thrombospondin-1	It is adhesive glycoprotein that mediates cell-to-cell and cell-to-matrix interactions.
15	*ZBTB4*	Zinc finger and BTB domain-containing protein 4	It is ranscriptional repressor with bimodal DNA-binding specificity.

## Discussion

In recent years, an increasing number of studies have drawn researchers’ attention to investigate the importance of miRNAs, which mainly participate in tumorigenesis and evolution. Profiling of miRNome (global miRNA expression levels) has become prevalent, and large amounts of miRNome data for a variety of cancer types are now available [16]. Hence, sufficient knowledge of the primary mechanisms of miRNA regulation could offer important insights into the effectiveness of carcinoma therapies. In the current study, cancer-related data from TCGA were analyzed to establish a regulatory network of miRNAs and mRNAs related to LUSC. Novel tumor markers have been used for diagnosing LUSC and developing effective treatments.

Seven miRNAs (hsa-miR-19a-3p, hsa-miR-126-5p, hsa-miR-556-3p, hsa-miR-671-5p, hsa-miR-937-3p, hsa-miR-4664-3p, and hsa-miR-4746-5p) were identified as independent prognostic factors in patients with LUSC via univariate Cox regression, multivariate Cox regression, and Kaplan-Meier analyses. An overall study based on a gene regulatory network in human hepatocellular carcinoma (HCC) revealed that abnormal regulation of the microRNA-19a/cyclin D1 axis has carcinogenic potential and poor prognosis [17]. Overexpression of miR-19a has also been observed in patients with malignant mesothelioma on pleural effusion cytology [18]. Christopher et al. [19] demonstrated that signature miRNAs are associated with oral squamous cell carcinoma and that miRNA-19a/b exerts a significant influence on the control of inflammatory responses. Additionally, the overexpression of miR-19a was associated with poor OS. A number of studies have reported that miR-126-5p is involved in the development of multiple carcinomas; it is highly expressed in ovarian carcinoma samples than in paired adjacent samples and was also found in ovarian carcinoma cell lines [20]. Meanwhile, the expression of miR-126-5p in human cervical cancer tumor tissue is abnormally downregulated compared with that in normal tissue [21], indicating that the inconsistent expression levels of this signature miRNA were related to the differences in cancer tissues or cells. In addition, an miR-126-5p/3p expression level beyond the median has been reported to be related to poor OS [22]. Our study found that miR-126-5p has low expression in LUSC, which is consistent with the findings of previous studies on miRNAs in lung cancer [23–24]. Studies on miR-556-3p have reported that miR-556-3p has low expression in endometrial tissues [25]. Wu [26] found that miR-556-3p is involved in regulating the sensitivity of circ-ABCB10 to lung cancer progression and resistance of lung cancer cells to cisplatin. MiR-671-5p is considered a cancer-related miRNA that is associated with cellular proliferation and invasion. Its overexpression in patients with colon cancer is associated with a poor prognosis [27]; meanwhile, the miR-671-5p expression in osteosarcoma tissues and cell lines is downregulated [28]. These contradictory results suggest that miR-671-5p can promote or inhibit various types of cancer, by mediating different targets. The expression of miR-671-5p in our study was elevated, whereas Harrison et al. [29] previously confirmed that miR-671-5p reduced the LUSC metastasis and identified miR-671-5p as an essential regulator of LUSC metastasis. MiR-937 has a significant function in the development of multiple illnesses. Yu et al. [30] reported that miR-937 was decreased in patients with gastric carcinoma, and further exploration demonstrated that miR-937 can regulate FOXL2 by suppressing the PI3K/AKT signaling pathway to inhibit the proliferation and metastasis of gastric carcinoma cells. The expression of miR-937-3p in breast carcinoma tissues and serum was higher than that in adjacent normal breast tissues, and the low expression of miR-937-3p could inhibit the proliferation, migration, and invasion of cancer cells. This study also confirmed that the overexpression of miR-937-3p was associated with poorer OS in patients with breast carcinoma [31]. The expression of miR-937 was higher in lung carcinoma tissues than in normal tissues, which can induce the proliferation of lung carcinoma cells through the regulation of INPP4B, whereas the downregulation of miR-937 reduces cell proliferation [32]. Previous studies have also shown that miR-4664-3p is significantly associated with the recurrence of small cell esophageal carcinoma [33], and their outcomes confirmed the overexpression of miR-4664 in patients with LUSC. Moreover, a previous study also demonstrated that miR-4746-5p is overexpressed in HPV16^+^ head and neck squamous cell carcinoma by regulating the epithelial to mesenchymal transition-related pathways, and that the high expression of miR-4746-5p is associated with good OS [34]. Additionally, miR-4746-5p is significantly associated with the prognosis of liver cancer [35]. Thus, these miRNAs have a significant function in the genesis and progression of tumors. Hence, further studies should be performed to investigate more important discoveries of these miRNAs and provide new meaningful insights into the establishment of novel approaches for cancer treatment. Risk scores based on prognostic characteristics can be used to distinguish low-risk patients from high-risk patients, the latter being significantly associated with poor outcomes. Unfortunately, although the TNM staging system is one of the most widely used prognostic indicators for LUSC, Cox analysis did not demonstrate the predictive power of TNM staging.

miRNAs are known to play key roles in a variety of biological processes associated with human diseases. A growing body of evidence suggests that miRNAs are closely associated with a variety of complex human diseases, such as diabetes [36]. osteoarthritic cartilage [37], Alzheimer’s disease [38], and cardiac hypertrophy [39]. For example, both miR-212 and mir-132 directly target anti-hypertrophic and pro-autophagy FoxO3 transcription factors, and the overexpression of these miRNAs results in the hyperactivation of pro-hypertrophic calcineurin/nuclear factor of activated T-cell signaling and impaired autophagy responses to starvation [39]. However, given the cost and complexity of biological experiments, computational methods that predict their potential associations with disease would be a useful complement. Therefore, an increasing number of researchers are committed to developing computational models for the identification of miRNA biomarkers for complex human diseases. The single biomarker model was first used by researchers, but a comprehensive model composed of multiple biomarkers has a higher predictive ability than a single biomarker model [40]. Problems frequently occur when constructing multiple biomarker models using traditional Cox regression models, such as a high fitting rate in the case of a large number of biomarkers. Then, the minimum absolute contraction and selection operator (LASSO) penalty Cox model was introduced to analyze the variable selection, which has been successfully applied to the creation of multiple biomarker models [41]. Zhang et al. [42] used LASSO-penalized multivariate survival models to predict the immune-associated miRNAs involved in the development of LUSC. In addition, more in-depth computational models can be used for the identification of miRNA biomarkers in human cancers. Xu et al. [43] proposed a prediction model to prioritize and identify the most promising miRNAs associated with multiple diseases by constructing an interaction network between miRNAs and target genes and between target genes and diseases. Chen et al. [44] proposed an ensemble of decision tree-based miRNA-disease association prediction calculation method. In the performance evaluation of the model, the AUC of fivefold cross-validation was 0.9192 +/−0.0009, which proved the reliability and stability of the model. In addition, some researchers have developed an MDHGI (Matrix Decompoeition and Herterogeneous Graph Inference) model. Experiments with MDHGI on different databases show that it has significant advantages over previous methods in missing one cross-validation and fivefold cross validation [45]. Most of these predicted miRNAs were confirmed by experimental literature. However, these computational models have not been applied to the identification of miRNAs in LUSC, and further studies are needed to determine how they can contribute to the identification these miRNAs.

Currently, a large number of studies have confirmed that miRNAs are closely correlated with the clinical characteristics of cancer. High expression of miR-25 is associated with lymph node metastasis and poor long-term survival in patients undergoing radical gastrectomy and systemic adjuvant chemotherapy [46]. In addition, the combination of three miRNAs (miR-125A-5p, miR-25, and miR-126) resulted in an AUC value of 0.94 for differentiating patients with early lung cancer from controls [47]. Li et al. [48] reported that high serum miR-25 levels were significantly associated with sex (p = 0.042), tumor stage (p = 0.014), and lymph node metastasis. However, in our study, perhaps due to the imbalance of the original data, the nomogram we developed could not adequately explain the correlation between the new miRNA and clinical characteristics.

Non-protein-coding RNAs (ncRNAs), including miRNAs, long ncRNAs (lncRNAs), and small interfering RNAs (siRNAs) control gene expression at different physiological levels. Increasing evidence indicates that lncRNAs are involved in a variety of cancer biological processes, such as epigenetic regulation, DNA damage, immune escape, and metabolic disorders [49]. lncRNAs and miRNAs regulate each other [50]; some researchers have developed a network distance analysis model for the prediction of the LNcrNA-mirNA association (NDALMA), which can calculate similar networks of lncRNAs and miRNAs, and their prediction results are reliable. The lncRNA-miRNA regulatory network plays an important role in tumor suppression and tumorigenesis [51]. Cao et al. [52] created lncRNA-miRNA-mRNA networks by downloading clinical information of HCC patients and RNA sequencing of miRNA, lncRNA, and mRNA data in TCGA, and identified new RNAs as biomarkers for HCC survival and prognosis. In the construction of IncRNA-miRNAs, a relatively complex algorithm is required to build the model. Zhang et al. [53] used a method based on a semi-supervised interaction group network to construct the LMI-INGI model to explore and predict the potential interaction between lncRNAs and miRNAs and achieved a high prediction effect (AUC = 0.8957). Based on the research experience of previous scholars, the miRNAs or lncRNAs involved in the development of LUSC can be identified using bioinformatics methods, and then mature algorithms and models can be used to predict the interactions of lncRNA-miRNAs. Further studies on the role of lncRNA-miRNAs in various physiological processes will help discover new biomarkers and treatments for LUSC.

Target genes and their related pathways were analyzed to further understand the functional roles of the seven miRNAs. KEGG pathway analysis revealed the primary enrichment of target genes in the Hippo signaling pathway as well as in HTLV-I infection. The HIPPO signaling cascade can be exploited by cancer cells to continue the development and progression of tumors. Lamar et al. [54] revealed that YAP overexpression in breast cancer and melanoma can enhance the migration capability of tumor cells, decrease central adhesion, develop the mesenchymal phenotype, and initiate the process of epithelial–mesenchymal transition. YAP, an important molecule in the Hippo signaling pathway, is widely activated in human malignant tumors. Similar to lung cancer, Gobbi et al. [55] demonstrated that LATS2, TAOK1, and NF2 act as Hippo pathway genes and are the primary determinants of the sensitivity of lung cancer cells to JQ1 pan-BETi. YAP-Hippo regulates MIEF1-related mitochondrial stress and activates the JNK pathway to promote the death of A549 lung cancer cells [56]. As a human retrovirus, HTLV-1 results in HTLV-1-related myelopathy/tropical spastic paraparesis and other inflammatory illnesses; HTLV-1 test is not a routine test, while approximately 5% of patients were found to be carriers of HTLV-1 in areas where HTLV-1 is endemic [57]. Yoneshima Y. et al. [58] reported that PD-1 inhibitors could have an effect on the response of the immune system to the virus, promoting the evolution of HTLV-1-related illnesses among patients with HTLV-1-positive NSCLC. Recently, by integrating bioinformatics and functional analyses, Song et al. [59] found that the hub genes and key miRNAs were primarily related to the cancer pathway, PI3K-Akt signaling pathway, and HTLV-1 infection in NSCLC. These results are consistent with our findings. The pathways of differential gene enrichment in LUSC patients obtained from GSEA software suggest that our main study is still focused on examining the p53 pathway as a follow-up experiment. These target genes contribute to the OS prediction or response to immunotherapy and could be potential biomarkers for treatment. Topology analyses in the PPI network were performed to further verify and screen the target genes. Fifteen genes were screened as hub genes, and a repeat survival analysis was conducted. The 15 hub genes, namely, ARC, CLVS2, ENMM5, FAM83D, HPRT1, HSPB8, ITGA2, LCLAT1, LONRF3, MBNL2, MED12L, NACC2, SLC6A8, THBS1, and ZBTB4, were significantly associated with the 10‐year survival rates of LUSC patients. However, further studies are required to verify this result.

This study identified a new characteristic miRNA based on the TCGA dataset and analyzed the prognosis of LUSC. These molecular signatures were validated through a series of independent experiments and functionality experiments. The limitations of this study are that the outcomes are not verified in clinical samples, and the comparatively small number of patients cannot offer a great statistical ability.

## Conclusion

Identification of new miRNA biomarkers was performed to predict the OS outcomes in squamous cellular lung carcinoma. The observations obtained from this study offer new insights into the establishment of novel approaches for reliable and precise carcinoma treatment.

## Supporting information

S1 Data (RAR)

S1 Table The differently expressed mRNAs in TCGA. (XLS)

S2 Table The differently expressed miRNAs in TCGA. (XLS)

S3 Table The results of univariate COX analysis. (XLS)

S4 Table Online databases to predicted 99 target genes of these seven miRNAs. (XLS)

S5 Table The results of GO analysis and KEGG analysis of target genes. (XLS)

## Abbreviations

miRNAs: microRNAs
LUSC: lung squamous cell carcinoma
TCGA: The Cancer Genome Atlas
OS: overall survival
RNA-seq: RNA sequencing
ROC: receiver operating characteristic
GO: Gene Ontology
KEGG: Kyoto Encyclopedia of Genes and Genomes
AUC: area under the curve
BP: biological processes
MF: molecular function
CC: cell component
HTLV-I: human T-cell leukemia virus type I
PPI: protein-protein interaction
global miRNA expression levels: Profiling of miRNome
HCC: hepatocellular carcinoma
MDHGI: Matrix Decompoeition and Herterogeneous Graph Inference
