# Supplementary material for: Identification of miRNA signature for predicting the prognostic biomarker of squamous cell lung carcinoma
Source: PLoS One. 2022 Mar 15;17(3):e0264645. doi: 10.1371/journal.pone.0264645 (PMC8923497; doi:10.1371/journal.pone.0264645)
Supplement: Attachment — Submitted filename: Response to Reviewers.docx [file pone.0264645.s007.docx]

Dear Editor,

Many thanks for your review our manuscript " Identification of miRNA signature for predicting prognostic biomarker in squamous cell lung carcinoma". Your comments about the paper give us many useful suggestions which help us correct some mistakes and think about some problems more deeply. Now we show the responses to each of the points raised in both the referees reports as follows.

Your Comments:

Q1: Please ensure that your manuscript meets PLOS ONE's style requirements, including those for file naming. The PLOS ONE style templates can be found at https://journals.plos.org/plosone/s/file?id=wjVg/PLOSOne_formatting_sample_main_body.pdf and https://journals.plos.org/plosone/s/file?id=ba62/PLOSOne_formatting_sample_title_authors_affiliations.pdf.

A1: Thank you. As your instructions, we had modified the manuscript to meet PLOS ONE's style requirements.

Q2: We suggest you thoroughly copyedit your manuscript for language usage, spelling, and grammar. If you do not know anyone who can help you do this, you may wish to consider employing a professional scientific editing service.

A2: Thank you for your good advice. We were aware of some language usage, spelling and grammar errors in the manuscript, so we asked Enpapers Company (http://www.enpapers.com/) to edit the article.

Q3: We note that you have stated that you will provide repository information for your data at acceptance. Should your manuscript be accepted for publication, we will hold it until you provide the relevant accession numbers or DOIs necessary to access your data. If you wish to make changes to your Data Availability statement, please describe these changes in your cover letter and we will update your Data Availability statement to reflect the information you provide.

A3: Thanks. We have reworked the data availability starement as “The datasets generated during the current study are publicly available from the following online databases: https://cancergenome.nih.gov/.”

Q4: Your ethics statement should only appear in the Methods section of your manuscript. If your ethics statement is written in any section besides the Methods, please move it to the Methods section and delete it from any other section. Please ensure that your ethics statement is included in your manuscript, as the ethics statement entered into the online submission form will not be published alongside your manuscript.

A4: Thanks for your reminding. We had moved the ethics statement in the Methods section of our manuscript.

Reviewer 1 Comments:

Q: The authors of this manuscript found 7 miRNAs and 15 hub genes which have close relation with the overall survival of lung squamous cell carcinoma patients using the R platform and series analytical tools. I hope the manuscript could be further strengthened by the following comments.

A: Thanks for your comments about our paper. As your instruction, we had done corresponding modification.

Q1: In the Introduction section, the detailed method of some relative researches which also identified the miRNA signature in squamous cell lung carcinoma should be declared.

A1: Thank you very much for your suggestion. According to your suggestion, we have added the methods of relevant studies that have identified the miRNA characteristics of LUSC.

Q2: In the Materials and methods section, the overall process should be shown as a graph.

A2: Thank you very much for your suggestion. We have added the flow chart (Fig 1) of this study in the section of Materials and Methods according to your suggestion.

Q3: In the Materials and methods section, the detailed process of univariate COX and multivariate COX regression analysis as well as Kaplan‐Meier curves should be provided.

A3: Thank you very much for your suggestion. We have added the detailed process of univariate COX and multivariate COX regression analysis as well as Kaplan Meier curve according to your suggestion.

Q4: In the Discussion section, this paper only discussed the 7 miRNAs which have been identified as independent prognostic factors of patients with LUSC and listed some literatures which can prove this conclusion. This way of validation is less persuasive since I’m not sure if there are other miRNAs which can also validated by literatures.

A4: Thank you for your good question. We all agree with you that there are indeed many miRNAs in the literature that are associated with LUSC prognosis. In our study, we used a variety of methods, such as R platform, univariate COX and multivariate COX and survival curve analysis, to screen the miRNAs contained in TCGA database layer by layer and finally determined these 7 miRNAs. These methods have been recognized by most scholars, so our results are convincing to some extent.

Q5: The reason why these seven miRNAs were chosen should be expressed more precisely.

A5: Thank you very much for your suggestion. As your instruction, we had done corresponding modification.

Q6: Could you discuss the recent trend of developing computational model for identification of the miRNA biomarker of human complex diseases as the future direction of your current research about miRNA biomarker identification of squamous cell lung carcinoma? Some important studies should be cited and discussed (PMIDs: 29939227, 29045685, 30142158, and 31329575).

A6: Thank you very much for your advice. We have added this part to the discussion section.

Q7: You should revise your English writing carefully and eliminate small errors in the paper to make the paper easier to understand.

A7: Thank you very much for your advice. We had done corresponding modification to make the paper easier to understand

Q8: It will provide the users helps to understand the method if the authors can present a high-quality flowchart figure for their methods.

A8: Thank you very much for your suggestion. We have added the flow chart of this study in the section of Materials and Methods according to your suggestion.

Reviewer 2 Comments:

Q: It is very important to identify microRNAs (miRNAs) involved in lung squamous cell carcinoma (LUSC) and exploit them as novel biomarkers or therapeutic targets. In the current manuscript, the authors downloaded LUSC-related miRNA and mRNA samples from TCGA. Then data had been preliminarily screened and pretreated, and the R platform and series analytical tools were used to identify specific and sensitive biomarkers. Besides, 7 miRNAs and 15 hub genes were eventually found to have close relation with the overall survival of LUSC patients. However, there are some problems to be further improved before acceptance for publication.

A: Thanks for your comments about our paper. As your instruction, we had done corresponding modification.

Q1: Literature review is somewhat incomplete in the introduction, especially about the progress of the bioinformatic analysis about the discovery of miRNA related with LUSC.

A1: Thank you very much for your suggestion. We have added the progress of bioinformatics analysis of LUSC-related mRNAS in the background preface according to your suggestion.

Q2: I suggest the authors should add a flowchart in the manuscript to show the process very well.

A2: Thank you very much for your suggestion. We have added the flow chart (Fig 1) of this study in the section of Materials and Methods according to your suggestion.

Q3: Could the authors add the data of other databases due to the AUC of both the training set and the testing set were no more than 0.7?

A3: Thank you very much for your question.

In this study, it is true that the AUC of both the training set and the test set is not greater than 0.7, but this result has been optimized after many tests.

Q4: Why the authors only perform the GO and KEGG pathway enrichment analyses? I recommend the authors should add the GSEA analysis.

A4: Thank you very much for your suggestion. We have added GSEA analysis to the article, the results showed in Fig 9A-9F.

Q5: I suggest that the authors should add a table to introduce the details of 7 miRNAs and 15 hub genes in the main body.

A5: Thank you very much for your suggestions. We have added the table of 15 hub genes in the paper, including the name, full name and function of genes (Table 2). However, as the main function of mRNAs aremainly involved in carcinoma progression and act as suppressors or promoters, there is no additional table attached.

Q6: I suggest the authors should discuss more about the meaning of their research in the last section, as they use several online tools.

A6: Thank you very much for your suggestions. We have made corresponding modifications in the manuscript.

Q7: Could the authors give some discussions whether their method and obtained result could be further used to predict LUSC-related ncRNAs? It could be considered as the future direction of their work. Some recommended studies are helpful (PMIDs: 33588070, 34232474, 34495484, 34329377; DOI: 10.1016/j.knosys.2019.105261).

A7: Thank you very much for your advice. We have added a section on predicting LUSC-related IncRNAs in the discussion section.

Q8: English expressions need to be edited more careful and more native, in this manuscript, there are some mistakes.

A8: Thank you very much for your advice. We have carefully edited the content of the article to make the English expression more fluent.
