# Supplementary material for: Identification of miRNA signature for predicting the prognostic biomarker of squamous cell lung carcinoma
Source: PLoS One. 2022 Mar 15;17(3):e0264645. doi: 10.1371/journal.pone.0264645 (PMC8923497; doi:10.1371/journal.pone.0264645)
Supplement: Attachment — Submitted filename: Response to Reviewers.docx [file pone.0264645.s008.docx]

Reviewer #1:

Q1: In the response letter of author, authors didn't give accurate revision position. Did yo want reviewer to search for the revision from the whole manuscript?

A1: We are very sorry for the inconvenience caused to you by the manuscript we modified before. This time we have colored all the changes we have made in response to the reviewers’ comments in our revised manuscript (Yellow indicates new content, and red indicates deleted content).

Q2: Paper writing weren't significantly improved.

A2: Thank you very much for your question. The manuscript's usage, spelling and grammar have been completely revised with the help of Editage, a professional editing service.

Q3: Important review about disease-miRNA association should be emphatically introduced (PMID: 29045685).

A3: Thank you very much for your advice. We have added this part to the discussion section. Lines 265 to 294 mainly introduced the correlation between diseases and miRNAs. The latest trends in developing computational models for miRNA biomarker identification of complex human diseases are also discussed, and the future direction of miRNA biomarker identification of squamous cell lung cancer is predicted.

Reviewer #2:

Q1: It is suggested to further explore the correlations between new miRNAs and clinical characteristics, and if possible, a nomogram should be designed to implement the scoring system.

A1: Thank you very much for your advice. Lines 295 to 303 in the discussion section of the manuscript discuss the correlation between miRNA and clinical characteristics reported in previous literatures. Due to the imbalance of our original data (the number of samples in the Normal group was small, and the number of samples in the cancer group was large, so the nomogram could not well explain the correlation between new miRNA and clinical features.

Q2: The authors should carefully answer my previous comment 7. The recommended study (PMIDs: 34232474, 34495484, 34329377; DOI: 10.1016/j.knosys.2019.105261) is ignored by the authors.

A2: I'm sorry for our previous work. Lines 304 through 322 of the discussion section of the manuscript discussed the prediction of LUSC-related ncRNAs. lncRNAs and miRNAs could regulate each other, and the lncRNA-miRNA regulatory network plays an important role in tumor suppression and tumorigenesis. At present, there are several models that can predict the potential association of miRNA-ncRNA, such as LMI-INGI model. Therefore, the miRNAs or ncRNAs involved in the development of LUSC can be identified using bioinformatics methods, and then mature algorithms and models can be used to predict the interactions of lncRNA-miRNAs.

Q3: The flowchart in Fig.1 is too simple to show the process very well, please improve it.

A3: Thank you very much for your advice. According to your suggestions, we revised and refined the flow chart of this study, as shown in Fig 1.

Q4: Some grammatical errors still appear in the manuscript. The authors should carefully check them.

A4: Thank you very much for your question. The manuscript's usage, spelling and grammar have been completely revised with the help of Editage (www.editage.com), a professional editing service.

Q5: The authors should highlight or color all the changes they have made in response to the reviewers’ comments in their revised manuscript. Please remove all markings about tracked changes.

A5: We are very sorry for the inconvenience caused to you by the manuscript we modified before. This time we have colored all the changes we have made in response to the reviewers’ comments in our revised manuscript (Yellow indicates new content, and red indicates deleted content).
