# Supplementary material for: Identification of miRNA signature for predicting the prognostic biomarker of squamous cell lung carcinoma
Source: PLoS One. 2022 Mar 15;17(3):e0264645. doi: 10.1371/journal.pone.0264645 (PMC8923497; doi:10.1371/journal.pone.0264645)
Supplement: Peer review history

# Peer review: Decision Letter 0

**DOI:** 10.1371/journal.pone.0264645.r001

**Authors:** Qi Zhao

26 Oct 2021

PONE-D-21-30577Identification of miRNA signature for predicting prognostic biomarker in squamous cell lung carcinomaPLOS ONE

Dear Dr. Jun Lyu,

Thank you for submitting your manuscript to PLOS ONE. After careful consideration, we feel that it has merit but does not fully meet PLOS ONE’s publication criteria as it currently stands. Therefore, we invite you to submit a revised version of the manuscript that addresses the points raised during the review process.

Please submit your revised manuscript by December 9, 2021. If you will need more time than this to complete your revisions, please reply to this message or contact the journal office at plosone@plos.org. When you're ready to submit your revision, log on to https://www.editorialmanager.com/pone/ and select the 'Submissions Needing Revision' folder to locate your manuscript file.

Please include the following items when submitting your revised manuscript:

- A rebuttal letter that responds to each point raised by the academic editor and reviewer(s). You should upload this letter as a separate file labeled 'Response to Reviewers'.
- A marked-up copy of your manuscript that highlights changes made to the original version. You should upload this as a separate file labeled 'Revised Manuscript with Track Changes'.
- An unmarked version of your revised paper without tracked changes. You should upload this as a separate file labeled 'Manuscript'.

If you would like to make changes to your financial disclosure, please include your updated statement in your cover letter. Guidelines for resubmitting your figure files are available below the reviewer comments at the end of this letter.

If applicable, we recommend that you deposit your laboratory protocols in protocols.io to enhance the reproducibility of your results. Protocols.io assigns your protocol its own identifier (DOI) so that it can be cited independently in the future. For instructions see: https://journals.plos.org/plosone/s/submission-guidelines#loc-laboratory-protocols. Additionally, PLOS ONE offers an option for publishing peer-reviewed Lab Protocol articles, which describe protocols hosted on protocols.io. Read more information on sharing protocols at https://plos.org/protocols?utm_medium=editorial-email&utm_source=authorletters&utm_campaign=protocols.

We look forward to receiving your revised manuscript.

Kind regards,

Qi Zhao

Academic Editor

PLOS ONE

Journal Requirements:

When submitting your revision, we need you to address these additional requirements.

1. Please ensure that your manuscript meets PLOS ONE's style requirements, including those for file naming. The PLOS ONE style templates can be found at 

https://journals.plos.org/plosone/s/file?id=wjVg/PLOSOne_formatting_sample_main_body.pdf and 

https://journals.plos.org/plosone/s/file?id=ba62/PLOSOne_formatting_sample_title_authors_affiliations.pdf

2. We suggest you thoroughly copyedit your manuscript for language usage, spelling, and grammar. If you do not know anyone who can help you do this, you may wish to consider employing a professional scientific editing service.  

Whilst you may use any professional scientific editing service of your choice, PLOS has partnered with both American Journal Experts (AJE) and Editage to provide discounted services to PLOS authors. Both organizations have experience helping authors meet PLOS guidelines and can provide language editing, translation, manuscript formatting, and figure formatting to ensure your manuscript meets our submission guidelines. To take advantage of our partnership with AJE, visit the AJE website (http://learn.aje.com/plos/) for a 15% discount off AJE services. To take advantage of our partnership with Editage, visit the Editage website (www.editage.com) and enter referral code PLOSEDIT for a 15% discount off Editage services.  If the PLOS editorial team finds any language issues in text that either AJE or Editage has edited, the service provider will re-edit the text for free.

Upon resubmission, please provide the following: 

● The name of the colleague or the details of the professional service that edited your manuscript

● A copy of your manuscript showing your changes by either highlighting them or using track changes (uploaded as a *supporting information* file)

● A clean copy of the edited manuscript (uploaded as the new *manuscript* file).

3. We note that you have stated that you will provide repository information for your data at acceptance. Should your manuscript be accepted for publication, we will hold it until you provide the relevant accession numbers or DOIs necessary to access your data. If you wish to make changes to your Data Availability statement, please describe these changes in your cover letter and we will update your Data Availability statement to reflect the information you provide.

4. Your ethics statement should only appear in the Methods section of your manuscript. If your ethics statement is written in any section besides the Methods, please move it to the Methods section and delete it from any other section. Please ensure that your ethics statement is included in your manuscript, as the ethics statement entered into the online submission form will not be published alongside your manuscript. 

[Note: HTML markup is below. Please do not edit.]

Reviewers' comments:

Reviewer's Responses to Questions

**Comments to the Author**

1. Is the manuscript technically sound, and do the data support the conclusions?

The manuscript must describe a technically sound piece of scientific research with data that supports the conclusions. Experiments must have been conducted rigorously, with appropriate controls, replication, and sample sizes. The conclusions must be drawn appropriately based on the data presented. 

Reviewer #1: Yes

Reviewer #2: Yes

**********

2. Has the statistical analysis been performed appropriately and rigorously? 

Reviewer #1: Yes

Reviewer #2: Yes

**********

3. Have the authors made all data underlying the findings in their manuscript fully available?

The PLOS Data policy requires authors to make all data underlying the findings described in their manuscript fully available without restriction, with rare exception (please refer to the Data Availability Statement in the manuscript PDF file). The data should be provided as part of the manuscript or its supporting information, or deposited to a public repository. For example, in addition to summary statistics, the data points behind means, medians and variance measures should be available. If there are restrictions on publicly sharing data—e.g. participant privacy or use of data from a third party—those must be specified.

Reviewer #1: Yes

Reviewer #2: Yes

**********

4. Is the manuscript presented in an intelligible fashion and written in standard English?

PLOS ONE does not copyedit accepted manuscripts, so the language in submitted articles must be clear, correct, and unambiguous. Any typographical or grammatical errors should be corrected at revision, so please note any specific errors here.

Reviewer #1: No

Reviewer #2: Yes

**********

5. Review Comments to the Author

Please use the space provided to explain your answers to the questions above. You may also include additional comments for the author, including concerns about dual publication, research ethics, or publication ethics. (Please upload your review as an attachment if it exceeds 20,000 characters)

Reviewer #1: The authors of this manuscript found 7 miRNAs and 15 hub genes which have close relation with the overall survival of lung squamous cell carcinoma patients using the R platform and series analytical tools. I hope the manuscript could be further strengthened by the following comments.

1. In the Introduction section, the detailed method of some relative researches which also identified the miRNA signature in squamous cell lung carcinoma should be declared.

2. In the Materials and methods section, the overall process should be shown as a graph.

3. In the Materials and methods section, the detailed process of univariate COX and multivariate COX regression analysis as well as Kaplan‐Meier curves should be provided.

4. In the Discussion section, this paper only discussed the 7 miRNAs which have been identified as independent prognostic factors of patients with LUSC and listed some literatures which can prove this conclusion. This way of validation is less persuasive since I’m not sure if there are other miRNAs which can also validated by literatures.

5. The reason why these seven miRNAs were chosen should be expressed more precisely.

6. Could you discuss the recent trend of developing computational model for identification of the miRNA biomarker of human complex diseases as the future direction of your current research about miRNA biomarker identification of squamous cell lung carcinoma? Some important studies should be cited and discussed (PMIDs: 29939227, 29045685, 30142158, and 31329575).

7. You should revise your English writing carefully and eliminate small errors in the paper to make the paper easier to understand.

8. It will provide the users helps to understand the method if the authors can present a high-quality flowchart figure for their methods.

Reviewer #2: It is very important to identify microRNAs (miRNAs) involved in lung squamous cell carcinoma (LUSC) and exploit them as novel biomarkers or therapeutic targets. In the current manuscript, the authors downloaded LUSC-related miRNA and mRNA samples from TCGA. Then data had been preliminarily screened and pretreated, and the R platform and series analytical tools were used to identify specific and sensitive biomarkers. Besides, 7 miRNAs and 15 hub genes were eventually found to have close relation with the overall survival of LUSC patients. However, there are some problems to be further improved before acceptance for publication.

1. Literature review is somewhat incomplete in the introduction, especially about the progress of the bioinformatic analysis about the discovery of miRNA related with LUSC.

2. I suggest the authors should add a flowchart in the manuscript to show the process very well.

3. Could the authors add the data of other databases due to the AUC of both the training set and the testing set were no more than 0.7?

4. Why the authors only perform the GO and KEGG pathway enrichment analyses? I recommend the authors should add the GSEA analysis.

5. I suggest that the authors should add a table to introduce the details of 7 miRNAs and 15 hub genes in the main body.

6. I suggest the authors should discuss more about the meaning of their research in the last section, as they use several online tools.

7. Could the authors give some discussions whether their method and obtained result could be further used to predict LUSC-related ncRNAs? It could be considered as the future direction of their work. Some recommended studies are helpful (PMIDs: 33588070, 34232474, 34495484, 34329377; DOI: 10.1016/j.knosys.2019.105261).

8. English expressions need to be edited more careful and more native, in this manuscript, there are some mistakes

**********

6. PLOS authors have the option to publish the peer review history of their article (what does this mean?). If published, this will include your full peer review and any attached files.

If you choose “no”, your identity will remain anonymous but your review may still be made public.

**Do you want your identity to be public for this peer review?** For information about this choice, including consent withdrawal, please see our Privacy Policy.

Reviewer #1: No

Reviewer #2: No

[NOTE: If reviewer comments were submitted as an attachment file, they will be attached to this email and accessible via the submission site. Please log into your account, locate the manuscript record, and check for the action link "View Attachments". If this link does not appear, there are no attachment files.]

While revising your submission, please upload your figure files to the Preflight Analysis and Conversion Engine (PACE) digital diagnostic tool, https://pacev2.apexcovantage.com/. PACE helps ensure that figures meet PLOS requirements. To use PACE, you must first register as a user. Registration is free. Then, login and navigate to the UPLOAD tab, where you will find detailed instructions on how to use the tool. If you encounter any issues or have any questions when using PACE, please email PLOS at figures@plos.org. Please note that Supporting Information files do not need this step.

# Author comment: Author response to Decision Letter 0

**DOI:** 10.1371/journal.pone.0264645.r002

25 Nov 2021

Dear Editor,

Many thanks for your review our manuscript " Identification of miRNA signature for predicting prognostic biomarker in squamous cell lung carcinoma". Your comments about the paper give us many useful suggestions which help us correct some mistakes and think about some problems more deeply. Now we show the responses to each of the points raised in both the referees reports as follows.

Your Comments:

Q1: Please ensure that your manuscript meets PLOS ONE's style requirements, including those for file naming. The PLOS ONE style templates can be found at https://journals.plos.org/plosone/s/file?id=wjVg/PLOSOne_formatting_sample_main_body.pdf and https://journals.plos.org/plosone/s/file?id=ba62/PLOSOne_formatting_sample_title_authors_affiliations.pdf. 

A1: Thank you. As your instructions, we had modified the manuscript to meet PLOS ONE's style requirements.

Q2: We suggest you thoroughly copyedit your manuscript for language usage, spelling, and grammar. If you do not know anyone who can help you do this, you may wish to consider employing a professional scientific editing service.

A2: Thank you for your good advice. We were aware of some language usage, spelling and grammar errors in the manuscript, so we asked Enpapers Company (http://www.enpapers.com/) to edit the article.

Q3: We note that you have stated that you will provide repository information for your data at acceptance. Should your manuscript be accepted for publication, we will hold it until you provide the relevant accession numbers or DOIs necessary to access your data. If you wish to make changes to your Data Availability statement, please describe these changes in your cover letter and we will update your Data Availability statement to reflect the information you provide.

A3: Thanks. We have reworked the data availability starement as “The datasets generated during the current study are publicly available from the following online databases: https://cancergenome.nih.gov/.”

Q4: Your ethics statement should only appear in the Methods section of your manuscript. If your ethics statement is written in any section besides the Methods, please move it to the Methods section and delete it from any other section. Please ensure that your ethics statement is included in your manuscript, as the ethics statement entered into the online submission form will not be published alongside your manuscript.

A4: Thanks for your reminding. We had moved the ethics statement in the Methods section of our manuscript.

Reviewer 1 Comments:

Q: The authors of this manuscript found 7 miRNAs and 15 hub genes which have close relation with the overall survival of lung squamous cell carcinoma patients using the R platform and series analytical tools. I hope the manuscript could be further strengthened by the following comments.

A: Thanks for your comments about our paper. As your instruction, we had done corresponding modification.

Q1: In the Introduction section, the detailed method of some relative researches which also identified the miRNA signature in squamous cell lung carcinoma should be declared.

A1: Thank you very much for your suggestion. According to your suggestion, we have added the methods of relevant studies that have identified the miRNA characteristics of LUSC.

Q2: In the Materials and methods section, the overall process should be shown as a graph.

A2: Thank you very much for your suggestion. We have added the flow chart (Fig 1) of this study in the section of Materials and Methods according to your suggestion.

Q3: In the Materials and methods section, the detailed process of univariate COX and multivariate COX regression analysis as well as Kaplan‐Meier curves should be provided.

A3: Thank you very much for your suggestion. We have added the detailed process of univariate COX and multivariate COX regression analysis as well as Kaplan Meier curve according to your suggestion.

Q4: In the Discussion section, this paper only discussed the 7 miRNAs which have been identified as independent prognostic factors of patients with LUSC and listed some literatures which can prove this conclusion. This way of validation is less persuasive since I’m not sure if there are other miRNAs which can also validated by literatures.

A4: Thank you for your good question. We all agree with you that there are indeed many miRNAs in the literature that are associated with LUSC prognosis. In our study, we used a variety of methods, such as R platform, univariate COX and multivariate COX and survival curve analysis, to screen the miRNAs contained in TCGA database layer by layer and finally determined these 7 miRNAs. These methods have been recognized by most scholars, so our results are convincing to some extent.

Q5: The reason why these seven miRNAs were chosen should be expressed more precisely.

A5: Thank you very much for your suggestion. As your instruction, we had done corresponding modification.

Q6: Could you discuss the recent trend of developing computational model for identification of the miRNA biomarker of human complex diseases as the future direction of your current research about miRNA biomarker identification of squamous cell lung carcinoma? Some important studies should be cited and discussed (PMIDs: 29939227, 29045685, 30142158, and 31329575).

A6: Thank you very much for your advice. We have added this part to the discussion section.

Q7: You should revise your English writing carefully and eliminate small errors in the paper to make the paper easier to understand.

A7: Thank you very much for your advice. We had done corresponding modification to make the paper easier to understand

Q8: It will provide the users helps to understand the method if the authors can present a high-quality flowchart figure for their methods.

A8: Thank you very much for your suggestion. We have added the flow chart of this study in the section of Materials and Methods according to your suggestion.

Reviewer 2 Comments:

Q: It is very important to identify microRNAs (miRNAs) involved in lung squamous cell carcinoma (LUSC) and exploit them as novel biomarkers or therapeutic targets. In the current manuscript, the authors downloaded LUSC-related miRNA and mRNA samples from TCGA. Then data had been preliminarily screened and pretreated, and the R platform and series analytical tools were used to identify specific and sensitive biomarkers. Besides, 7 miRNAs and 15 hub genes were eventually found to have close relation with the overall survival of LUSC patients. However, there are some problems to be further improved before acceptance for publication.

A: Thanks for your comments about our paper. As your instruction, we had done corresponding modification.

Q1: Literature review is somewhat incomplete in the introduction, especially about the progress of the bioinformatic analysis about the discovery of miRNA related with LUSC.

A1: Thank you very much for your suggestion. We have added the progress of bioinformatics analysis of LUSC-related mRNAS in the background preface according to your suggestion.

Q2: I suggest the authors should add a flowchart in the manuscript to show the process very well.

A2: Thank you very much for your suggestion. We have added the flow chart (Fig 1) of this study in the section of Materials and Methods according to your suggestion.

Q3: Could the authors add the data of other databases due to the AUC of both the training set and the testing set were no more than 0.7?

A3: Thank you very much for your question.

In this study, it is true that the AUC of both the training set and the test set is not greater than 0.7, but this result has been optimized after many tests.

Q4: Why the authors only perform the GO and KEGG pathway enrichment analyses? I recommend the authors should add the GSEA analysis.

A4: Thank you very much for your suggestion. We have added GSEA analysis to the article, the results showed in Fig 9A-9F. 

Q5: I suggest that the authors should add a table to introduce the details of 7 miRNAs and 15 hub genes in the main body.

A5: Thank you very much for your suggestions. We have added the table of 15 hub genes in the paper, including the name, full name and function of genes (Table 2). However, as the main function of mRNAs aremainly involved in carcinoma progression and act as suppressors or promoters, there is no additional table attached.

Q6: I suggest the authors should discuss more about the meaning of their research in the last section, as they use several online tools.

A6: Thank you very much for your suggestions. We have made corresponding modifications in the manuscript.

Q7: Could the authors give some discussions whether their method and obtained result could be further used to predict LUSC-related ncRNAs? It could be considered as the future direction of their work. Some recommended studies are helpful (PMIDs: 33588070, 34232474, 34495484, 34329377; DOI: 10.1016/j.knosys.2019.105261).

A7: Thank you very much for your advice. We have added a section on predicting LUSC-related IncRNAs in the discussion section.

Q8: English expressions need to be edited more careful and more native, in this manuscript, there are some mistakes.

A8: Thank you very much for your advice. We have carefully edited the content of the article to make the English expression more fluent.

Attachment Submitted filename: Response to Reviewers.docx Click here for additional data file.

# Peer review: Decision Letter 1

**DOI:** 10.1371/journal.pone.0264645.r003

**Authors:** Qi Zhao

9 Dec 2021

PONE-D-21-30577R1Identification of miRNA signature for predicting prognostic biomarker in squamous cell lung carcinomaPLOS ONE

Dear Prof. Jun Lyu,

Thank you for submitting your manuscript to PLOS ONE. After careful consideration, we feel that it has merit but does not fully meet PLOS ONE’s publication criteria as it currently stands. Therefore, we invite you to submit a revised version of the manuscript that addresses the points raised during the review process.

Please submit your revised manuscript by Jan 23 2022 11:59PM. If you will need more time than this to complete your revisions, please reply to this message or contact the journal office at plosone@plos.org. When you're ready to submit your revision, log on to https://www.editorialmanager.com/pone/ and select the 'Submissions Needing Revision' folder to locate your manuscript file.

Please include the following items when submitting your revised manuscript:

- A rebuttal letter that responds to each point raised by the academic editor and reviewer(s). You should upload this letter as a separate file labeled 'Response to Reviewers'.
- A marked-up copy of your manuscript that highlights changes made to the original version. You should upload this as a separate file labeled 'Revised Manuscript with Track Changes'.
- An unmarked version of your revised paper without tracked changes. You should upload this as a separate file labeled 'Manuscript'.

If you would like to make changes to your financial disclosure, please include your updated statement in your cover letter. Guidelines for resubmitting your figure files are available below the reviewer comments at the end of this letter.

If applicable, we recommend that you deposit your laboratory protocols in protocols.io to enhance the reproducibility of your results. Protocols.io assigns your protocol its own identifier (DOI) so that it can be cited independently in the future. For instructions see: https://journals.plos.org/plosone/s/submission-guidelines#loc-laboratory-protocols. Additionally, PLOS ONE offers an option for publishing peer-reviewed Lab Protocol articles, which describe protocols hosted on protocols.io. Read more information on sharing protocols at https://plos.org/protocols?utm_medium=editorial-email&utm_source=authorletters&utm_campaign=protocols.

We look forward to receiving your revised manuscript.

Kind regards,

Qi Zhao

Academic Editor

PLOS ONE

[Note: HTML markup is below. Please do not edit.]

Reviewers' comments:

Reviewer's Responses to Questions

**Comments to the Author**

1. If the authors have adequately addressed your comments raised in a previous round of review and you feel that this manuscript is now acceptable for publication, you may indicate that here to bypass the “Comments to the Author” section, enter your conflict of interest statement in the “Confidential to Editor” section, and submit your "Accept" recommendation.

Reviewer #1: (No Response)

Reviewer #2: (No Response)

**********

2. Is the manuscript technically sound, and do the data support the conclusions?

The manuscript must describe a technically sound piece of scientific research with data that supports the conclusions. Experiments must have been conducted rigorously, with appropriate controls, replication, and sample sizes. The conclusions must be drawn appropriately based on the data presented. 

Reviewer #1: (No Response)

Reviewer #2: (No Response)

**********

3. Has the statistical analysis been performed appropriately and rigorously? 

Reviewer #1: (No Response)

Reviewer #2: (No Response)

**********

4. Have the authors made all data underlying the findings in their manuscript fully available?

The PLOS Data policy requires authors to make all data underlying the findings described in their manuscript fully available without restriction, with rare exception (please refer to the Data Availability Statement in the manuscript PDF file). The data should be provided as part of the manuscript or its supporting information, or deposited to a public repository. For example, in addition to summary statistics, the data points behind means, medians and variance measures should be available. If there are restrictions on publicly sharing data—e.g. participant privacy or use of data from a third party—those must be specified.

Reviewer #1: (No Response)

Reviewer #2: (No Response)

**********

5. Is the manuscript presented in an intelligible fashion and written in standard English?

PLOS ONE does not copyedit accepted manuscripts, so the language in submitted articles must be clear, correct, and unambiguous. Any typographical or grammatical errors should be corrected at revision, so please note any specific errors here.

Reviewer #1: (No Response)

Reviewer #2: (No Response)

**********

6. Review Comments to the Author

Please use the space provided to explain your answers to the questions above. You may also include additional comments for the author, including concerns about dual publication, research ethics, or publication ethics. (Please upload your review as an attachment if it exceeds 20,000 characters)

Reviewer #1: 1. In the response letter of author, authors didn't give accurate revision position. Did yo want reviewer to search for the revision from the whole manuscript?

2. Paper writing weren't significantly improved.

3. Important review about disease-miRNA association should be emphatically introduced (PMID: 29045685).

Reviewer #2: 1. It is suggested to further explore the correlations between new miRNAs and clinical characteristics, and if possible, a nomogram should be designed to implement the scoring system.

2. The authors should carefully answer my previous comment 7. The recommended study (PMIDs: 34232474, 34495484, 34329377; DOI: 10.1016/j.knosys.2019.105261) is ignored by the authors.

3. The flowchart in Fig.1 is too simple to show the process very well, please improve it.

4. Some grammatical errors still appear in the manuscript. The authors should carefully check them.

5. The authors should highlight or color all the changes they have made in response to the reviewers’ comments in their revised manuscript. Please remove all markings about tracked changes.

**********

7. PLOS authors have the option to publish the peer review history of their article (what does this mean?). If published, this will include your full peer review and any attached files.

If you choose “no”, your identity will remain anonymous but your review may still be made public.

**Do you want your identity to be public for this peer review?** For information about this choice, including consent withdrawal, please see our Privacy Policy.

Reviewer #1: No

Reviewer #2: No

[NOTE: If reviewer comments were submitted as an attachment file, they will be attached to this email and accessible via the submission site. Please log into your account, locate the manuscript record, and check for the action link "View Attachments". If this link does not appear, there are no attachment files.]

While revising your submission, please upload your figure files to the Preflight Analysis and Conversion Engine (PACE) digital diagnostic tool, https://pacev2.apexcovantage.com/. PACE helps ensure that figures meet PLOS requirements. To use PACE, you must first register as a user. Registration is free. Then, login and navigate to the UPLOAD tab, where you will find detailed instructions on how to use the tool. If you encounter any issues or have any questions when using PACE, please email PLOS at figures@plos.org. Please note that Supporting Information files do not need this step.

# Author comment: Author response to Decision Letter 1

**DOI:** 10.1371/journal.pone.0264645.r004

5 Feb 2022

Reviewer #1: 

Q1: In the response letter of author, authors didn't give accurate revision position. Did yo want reviewer to search for the revision from the whole manuscript?

A1: We are very sorry for the inconvenience caused to you by the manuscript we modified before. This time we have colored all the changes we have made in response to the reviewers’ comments in our revised manuscript (Yellow indicates new content, and red indicates deleted content). 

Q2: Paper writing weren't significantly improved.

A2: Thank you very much for your question. The manuscript's usage, spelling and grammar have been completely revised with the help of Editage, a professional editing service.

Q3: Important review about disease-miRNA association should be emphatically introduced (PMID: 29045685).

A3: Thank you very much for your advice. We have added this part to the discussion section. Lines 265 to 294 mainly introduced the correlation between diseases and miRNAs. The latest trends in developing computational models for miRNA biomarker identification of complex human diseases are also discussed, and the future direction of miRNA biomarker identification of squamous cell lung cancer is predicted.

Reviewer #2: 

Q1: It is suggested to further explore the correlations between new miRNAs and clinical characteristics, and if possible, a nomogram should be designed to implement the scoring system.

A1: Thank you very much for your advice. Lines 295 to 303 in the discussion section of the manuscript discuss the correlation between miRNA and clinical characteristics reported in previous literatures. Due to the imbalance of our original data (the number of samples in the Normal group was small, and the number of samples in the cancer group was large, so the nomogram could not well explain the correlation between new miRNA and clinical features.

Q2: The authors should carefully answer my previous comment 7. The recommended study (PMIDs: 34232474, 34495484, 34329377; DOI: 10.1016/j.knosys.2019.105261) is ignored by the authors.

A2: I'm sorry for our previous work. Lines 304 through 322 of the discussion section of the manuscript discussed the prediction of LUSC-related ncRNAs. lncRNAs and miRNAs could regulate each other, and the lncRNA-miRNA regulatory network plays an important role in tumor suppression and tumorigenesis. At present, there are several models that can predict the potential association of miRNA-ncRNA, such as LMI-INGI model. Therefore, the miRNAs or ncRNAs involved in the development of LUSC can be identified using bioinformatics methods, and then mature algorithms and models can be used to predict the interactions of lncRNA-miRNAs.

Q3: The flowchart in Fig.1 is too simple to show the process very well, please improve it.

A3: Thank you very much for your advice. According to your suggestions, we revised and refined the flow chart of this study, as shown in Fig 1.

Q4: Some grammatical errors still appear in the manuscript. The authors should carefully check them.

A4: Thank you very much for your question. The manuscript's usage, spelling and grammar have been completely revised with the help of Editage (www.editage.com), a professional editing service.

Q5: The authors should highlight or color all the changes they have made in response to the reviewers’ comments in their revised manuscript. Please remove all markings about tracked changes.

A5: We are very sorry for the inconvenience caused to you by the manuscript we modified before. This time we have colored all the changes we have made in response to the reviewers’ comments in our revised manuscript (Yellow indicates new content, and red indicates deleted content).

Attachment Submitted filename: Response to Reviewers.docx Click here for additional data file.

# Peer review: Decision Letter 2

**DOI:** 10.1371/journal.pone.0264645.r005

**Authors:** Khushboo Irshad

15 Feb 2022

Identification of miRNA signature for predicting prognostic biomarker in squamous cell lung carcinoma

PONE-D-21-30577R2

Dear Dr. Jun Lyu,

We’re pleased to inform you that your manuscript has been judged scientifically suitable for publication and will be formally accepted for publication once it meets all outstanding technical requirements.

Within one week, you’ll receive an e-mail detailing the required amendments. When these have been addressed, you’ll receive a formal acceptance letter and your manuscript will be scheduled for publication.

An invoice for payment will follow shortly after the formal acceptance. To ensure an efficient process, please log into Editorial Manager at http://www.editorialmanager.com/pone/, click the 'Update My Information' link at the top of the page, and double check that your user information is up-to-date. If you have any billing related questions, please contact our Author Billing department directly at authorbilling@plos.org.

If your institution or institutions have a press office, please notify them about your upcoming paper to help maximize its impact. If they’ll be preparing press materials, please inform our press team as soon as possible -- no later than 48 hours after receiving the formal acceptance. Your manuscript will remain under strict press embargo until 2 pm Eastern Time on the date of publication. For more information, please contact onepress@plos.org.

Kind regards,

Khushboo Irshad, Ph.D.

Academic Editor

PLOS ONE

Additional Editor Comments (optional):

Reviewers' comments:

Reviewer's Responses to Questions

**Comments to the Author**

1. If the authors have adequately addressed your comments raised in a previous round of review and you feel that this manuscript is now acceptable for publication, you may indicate that here to bypass the “Comments to the Author” section, enter your conflict of interest statement in the “Confidential to Editor” section, and submit your "Accept" recommendation.

Reviewer #1: (No Response)

**********

2. Is the manuscript technically sound, and do the data support the conclusions?

The manuscript must describe a technically sound piece of scientific research with data that supports the conclusions. Experiments must have been conducted rigorously, with appropriate controls, replication, and sample sizes. The conclusions must be drawn appropriately based on the data presented. 

Reviewer #1: (No Response)

**********

3. Has the statistical analysis been performed appropriately and rigorously? 

Reviewer #1: (No Response)

**********

4. Have the authors made all data underlying the findings in their manuscript fully available?

The PLOS Data policy requires authors to make all data underlying the findings described in their manuscript fully available without restriction, with rare exception (please refer to the Data Availability Statement in the manuscript PDF file). The data should be provided as part of the manuscript or its supporting information, or deposited to a public repository. For example, in addition to summary statistics, the data points behind means, medians and variance measures should be available. If there are restrictions on publicly sharing data—e.g. participant privacy or use of data from a third party—those must be specified.

Reviewer #1: (No Response)

**********

5. Is the manuscript presented in an intelligible fashion and written in standard English?

PLOS ONE does not copyedit accepted manuscripts, so the language in submitted articles must be clear, correct, and unambiguous. Any typographical or grammatical errors should be corrected at revision, so please note any specific errors here.

Reviewer #1: (No Response)

**********

6. Review Comments to the Author

Please use the space provided to explain your answers to the questions above. You may also include additional comments for the author, including concerns about dual publication, research ethics, or publication ethics. (Please upload your review as an attachment if it exceeds 20,000 characters)

Reviewer #1: (No Response)

**********

7. PLOS authors have the option to publish the peer review history of their article (what does this mean?). If published, this will include your full peer review and any attached files.

If you choose “no”, your identity will remain anonymous but your review may still be made public.

**Do you want your identity to be public for this peer review?** For information about this choice, including consent withdrawal, please see our Privacy Policy.

Reviewer #1: No

# Editor report: Acceptance letter

**DOI:** 10.1371/journal.pone.0264645.r006

**Authors:** Khushboo Irshad

3 Mar 2022

PONE-D-21-30577R2 

Identification of miRNA signature for predicting the prognostic biomarker of squamous cell lung carcinoma 

Dear Dr. Lyu:

I'm pleased to inform you that your manuscript has been deemed suitable for publication in PLOS ONE. Congratulations! Your manuscript is now with our production department. 

If your institution or institutions have a press office, please let them know about your upcoming paper now to help maximize its impact. If they'll be preparing press materials, please inform our press team within the next 48 hours. Your manuscript will remain under strict press embargo until 2 pm Eastern Time on the date of publication. For more information please contact onepress@plos.org.

If we can help with anything else, please email us at plosone@plos.org. 

Thank you for submitting your work to PLOS ONE and supporting open access. 

Kind regards, 

PLOS ONE Editorial Office Staff

on behalf of

Dr. Khushboo Irshad 

Academic Editor

PLOS ONE
